# Chemometric insights into *Lactiplantibacillus plantarum* effects on onion (*Allium cepa* L.) metabolism and antidiabetic activity under cadmium stress

**DOI:** 10.1038/s41598-026-67251-0

**Published:** 2026-09-05

**Authors:** Salwa M. Abdel Rahman, Nancy M. El Halfawy, Rahma S. R. Mahrous

**Affiliations:** 1 Department of Botany and Microbiology, Faculty of Science, Alexandria University, Alexandria, Egypt; 2 Department of Pharmacognosy, Faculty of Pharmacy, Alexandria University, 1 El-Khartoum Square-Azarita, Alexandria, 21521 Egypt

**Keywords:** *Allium cepa* L., *Lactiplantibacillus plantarum* 10CH, Cadmium stress, Heavy metal, Antidiabetic activity, α-glucosidase inhibitory activity, Biochemistry, Biotechnology, Microbiology, Plant sciences

## Abstract

Cadmium (Cd) is a toxic heavy metal that causes severe physiological damage in plants, inhibiting growth and ultimately reducing crop yield. Lactic acid bacteria regulate Cd availability through bioaccumulation and biosorption. This study evaluated the Cd tolerance of *Lactiplantibacillus plantarum* 10CH by determining its survival capacity under Cd stress and its potential to mitigate Cd-induced stress in onion (*Allium cepa* L.). The bacterial strain tolerated Cd concentrations up to 100 µM, and whole-genome sequencing identified genes involved in Cd biosorption, accumulation, and efflux. Exposure of onion to increasing CdCl_2_ concentrations significantly reduced root and shoot biomass. Inoculation with *Lb. plantarum* 10CH alleviated Cd stress at 100 µM, enhancing root and shoot biomass, reducing Cd accumulation, lowering oxidative damage markers, and stimulating antioxidant enzyme activities. Metabolic profiling revealed that Cd stress significantly reduced primary metabolites and amino acids, particularly at 100 µM, while bacterial inoculation restored key amino acids and peptides, including arginine, tyrosine, and glutamic acid. Chemometric analysis using unsupervised (PCA) and supervised (OPLS-DA) models revealed clear metabolite variation among untreated, Cd-stressed, and bacterial inoculated Cd-stressed onion leaves. Furthermore, leaf extracts exhibited α-glucosidase inhibitory activity, with the highest activity in control plants (IC_50_ = 425.2 ± 0.5 µg/mL). Cd-stressed plants showed moderate antidiabetic activity, which was significantly reduced by bacterial inoculation. Overall, these findings demonstrate that *Lb. plantarum* 10CH can survive under Cd stress and alleviates Cd-induced stress in onion, highlighting its potential as a bioinoculant to mitigate heavy metal stress.

**Supplementary Information:**

The online version contains supplementary material available at https://doi.org/10.1038/s41598-026-67251-0.

## Introduction

Environmental pollution resulting from heavy metal accumulation has become a significant concern owing to increasing urbanization and extensive anthropogenic activities, such as the combustion of fossil fuels^1^. Cadmium (Cd) is a toxic heavy metal that causes major health concerns even at low concentrations. It is one of the most concerning environmental pollutants, known for its high accumulation in soil and its detrimental effects on humans, animals, and plants^2^. Soil Cd contamination primarily arises from anthropogenic sources, including mining, smelting, industrial emissions, and the use of phosphate fertilizers. Additional inputs come from atmospheric deposition associated with fossil fuel combustion, and from sewage sludge application, both of which significantly increase Cd concentrations in soils^3,4^. Cadmium does not play a biological role in plant metabolic functions^5^. Its uptake by plants even at low concentrations leads to accumulation in various tissues, disruption of nitrogen metabolism, and inhibition of plant growth^6^. Cadmium impairs nutrient uptake and homeostasis by disrupting the absorption and transport of essential minerals, including iron (Fe), zinc (Zn), calcium (Ca), magnesium (Mg), and manganese (Mn)^7^. It also inhibits photosynthetic performance through reduced chlorophyll production, damage to chloroplast ultrastructure, and interference with the electron transport chain^8^. Furthermore, Exposure to Cd triggers the overproduction of reactive oxygen species (ROS), resulting in oxidative stress and causing lipid peroxidation, protein oxidation, membrane deterioration, and DNA damage^9^.

Moreover, Plants grown in cadmium-contaminated soils serve as a major pathway for cadmium entry into the food chain, thereby posing health risks to both animals and humans^2^. Furthermore, this heavy metal and its compounds have been classified as carcinogenic to humans by the International Agency for Research on Cancer (IARC)^1^.

Various approaches have been utilized to reduce Cd availability in soil, including the addition of organic amendments such as compost and biochar^10^, as well as soil pH adjustment through liming, which significantly decreases Cd mobility^11^. Moreover, supplying adequate levels of essential nutrients has been shown to reduce Cd uptake by plants^12^. Biological approaches have also emerged as promising strategies for mitigating Cd toxicity in plants. Inoculation with arbuscular mycorrhizal fungi has been reported to enhance the production of organic acids and phytohormones, thereby alleviating Cd-induced stress in plants^13^. Plant growth-promoting rhizobacteria play a crucial role in mitigating Cd toxicity in plants^14^. They alleviate the toxic effect of Cd via several mechanisms, including Cd chelation via the production of siderophores and organic acids that form stable complexes with reduced bioavailability^15^, enhancing plant stress tolerance via production of phytohormones^16^, and stimulation of antioxidant defense systems^17^.

Lactic acid bacteria (LAB) are safe, non-toxic bacterial strains with potential applications in soil remediation^18^. They enhance plant tolerance to heavy metals through mechanisms such as extracellular biosorption and intracellular bioaccumulation^19^. Extracellular biosorption is a metabolism-independent process wherein metal cations are sequestered through electrostatic interactions with anionic functional groups present on the bacterial cell envelope and within the exopolysaccharides (EPS) secreted by LAB. Through ion exchange, electrostatic attraction, and surface complexation, these functional groups immobilize metals such as Cd^2+^, Pb^2+^, and Ni^2+20,21^. By contrast, intracellular bioaccumulation is an active, energy-dependent process in which metal ions cross the plasma membrane into the cytoplasm, where they are bound by intracellular metal-binding proteins and peptides that lower their reactivity and cellular toxicity^22^. These physiological responses are regulated by genetic resistance determinants that function via two distinct pathways. One pathway comprises enzymatic biotransformation, which converts highly toxic metal species into less noxious forms^21^. The other involves active efflux systems, whereby cytoplasmic metal concentrations are maintained below toxic thresholds through membrane-bound transporters, specifically P-type ATPases and Resistance-Nodulation-Division (RND) transporters that pump excess cations out of the cell. This mechanism is extensively documented among metal-resistant, plant-associated bacteria^23^.

*Allium cepa* L. (onion), a well-known member of the Amaryllidaceae family, is widely recognized for its food and medicinal uses across the globe^24^. It is valued not only as a key flavoring agent in various cuisines but also for its significant nutritional content. According to the Food and Agriculture Organization (FAO), onion ranks among the 15 most extensively cultivated crops worldwide^25^. Onion possesses many health benefits, such as antioxidant, anti-inflammatory, and antimicrobial activities, particularly against yeasts and molds^26^. Furthermore, antiviral effects have been observed against pathogens such as adenovirus, herpes simplex virus type I, and HIV. Regular onion consumption has also been associated with a reduced risk of several cancers, including liver, breast, esophageal, pancreatic, and gastric cancers. In addition, it exhibits antidiabetic, antihyperlipidemic, antiplatelet, and hepatoprotective properties^27^. These therapeutic effects are largely attributed to the diverse phytoconstituents present in both the bulb extract and essential oil. Onion is a rich source of bioactive compounds of various classes, including flavonoids, phenolic acids, anthocyanins, saponins, peptides, and amino acids^26,27^. Notably, organosulfur compounds, which are abundant in onions and contribute to their characteristic aroma, have been shown to possess potent antioxidant, antimicrobial, and anticancer properties^24,27^.

The cytotoxic effects of cadmium on *Allium cepa* L. have been well documented^28,29^. Exposure to high levels of Cd was correlated with significant reductions in mineral content across various plant tissues, including roots, bulbs, and leaves^30^. This adversely affects the growth and yield of this economically important crop, thereby posing indirect risks to human health. To the best of our knowledge, no study has yet evaluated the Cd-tolerance and Cd-remediation potential of *Lactiplantibacillus plantarum* 10CH or its effectiveness in mitigating cadmium-induced stress in onion (*Allium cepa* L.). Consequently, this study aimed to assess the efficacy of *Lb. plantarum* 10CH as a biological agent for alleviating cadmium stress in *A. cepa* L. and its overall effect on the plant’s antidiabetic activity. First, whole-genome sequencing (WGS) data of *Lb. plantarum* 10CH were analyzed using computational pipelines to identify genes associated with heavy metal uptake and stress response mechanisms, and establish the mechanistic basis underlying the bacterial strain’s potential role in Cd tolerance. Subsequently, the effects of *Lb. plantarum* 10CH on plant growth, antioxidant enzyme activities, and the phytochemical composition of onion shoots exposed to Cd stress were evaluated. Furthermore, the influence of Cd stress and bacterial inoculation on the antidiabetic potential of onion leaves was investigated in vitro using an α-glucosidase inhibitory assay. Finally, chemometric analysis, including PCA and OPLS-DA, was employed to explore metabolite variation among untreated, Cd-stressed, and bacterial inoculated Cd-stressed onion leaves. Besides, an OPLS model was used to correlate metabolite profiles with antidiabetic activity, providing insight into the relationship between Cd-induced metabolic shifts and antidiabetic bioactivity.

## Materials and methods

### Bacterial strains and in silico prediction of heavy metal tolerance genes

*Lactiplantibacillus plantarum* 10CH (NCBI Accession no. CP023728) was activated by culturing in De Man–Rogosa–Sharpe broth medium (MRS; HiMedia, India) at 30 °C for 24 h and used in further investigation. To identify heavy-metal tolerance genes in the genome sequence, the Bacterial and Viral Bioinformatics Resource Center (BV-BRC; https://www.bvbrc.org/) platform was used. For homology-based identification, all previously predicted coding sequences (CDSs) in the genome were translated, and the resulting protein sequences were subjected to Basic Local Alignment Search Tool (BLASTp) analyses against the National Center for Biotechnology Information (NCBI) database (https://blast.ncbi.nlm.nih.gov/).

### Tolerance of *Lb. plantarum* 10CH to cadmium

Heavy metal tolerance of *Lb. plantarum* 10CH was evaluated in vitro in accordance with a previously reported method^31^. Test tubes containing 15 mL of MRS broth were supplemented with cadmium chloride (CdCl_2_) at final concentrations of 0 (control), 50, 100, and 200 µM. Each MRS tube was inoculated with 1.0% (v/v) of a freshly prepared culture of *Lb. plantarum* 10CH, adjusted to 10^8^ CFU/mL. The cultures were incubated statically at 30 °C for 48 h. Bacterial growth was monitored by measuring the optical density at 600 nm using a spectrophotometer (Jenway 6305, UK). Tolerance to cadmium was defined as visible bacterial growth in the presence of CdCl_2_ relative to the control culture without cadmium. Following incubation, the viability of cells was further confirmed by cultivating the treated *Lb. plantarum* 10CH cells onto MRS agar plates and incubating at 30 °C for 48 h. The presence of visible colonies on MRS agar was interpreted as confirmation of cell viability, whereas the absence of growth indicated lethal cadmium toxicity. All experiments were performed in replicates.

### Inoculation of *Allium cepa* L. seeds with *Lb. plantarum* 10CH

Onion seeds (*Allium cepa* L., cv. Itali) were obtained from the Seed Department, Agriculture Directorate in Alexandria (Egypt). Onion seeds were sterilized by soaking for 2 min in 4.0% sodium hypochlorite solution, followed by numerous washes in distilled water. The sterilized onion seeds were inoculated by immersing them for 2 h at room temperature in a bacterial suspension of *Lb. plantarum* 10CH (10^8^ CFU/mL) supplemented with 2.0% (w/v) sucrose^14^.

### Cultivation of *Allium cepa* L. seeds under cadmium stress

A pot experiment was performed in the greenhouse at the Faculty of Science, Alexandria University (Egypt). The experimental design comprised seven treatments: 0 = control untreated; 50 *N* = 50 µM Cd without bacterial inoculation; 50I = 50 µM Cd with bacterial inoculation; 100 *N* = 100 µM Cd without inoculation; 100I = 100 µM Cd with bacterial inoculation; 200 *N* = 200 µM Cd without inoculation; and 200I = 200 µM Cd with bacterial inoculation. After 6 weeks of cultivation, both inoculated and non-inoculated plants were irrigated daily with 1 mL of CdCl_2_ of different concentrations (50, 100, and 200 µM) added to the irrigation water^32^. Following six days of Cd treatment, seedlings were harvested, thoroughly washed, and used to determine root and shoot dry weights and to assess Cd accumulation in plant tissues. The pot experiment was performed in triplicate under controlled conditions (20 ± 2 °C, 75 ± 2% relative humidity, and a 14/10 h light/dark photoperiod).

### Analysis of Cd content in soil and *Allium cepa* L

Soil subsamples were collected from the 50, 100, and 200 µM Cd treatments. The soil samples were air-dried, subjected to strong acid digestion, and the resulting solutions were analyzed for Cd concentration. Onion roots and leaves were oven-dried and digested according to the method of Jones et al.^28^. Cadmium concentrations in the soil and plant digests were determined using an atomic absorption spectrophotometer (Perkin-Elmer 100B, Waltham, MA, USA) following the method of Isaac^29^.

### Calculation of the Cd bio-concentration factor (BCF)

BCF is defined as the ratio of the concentration of the metal in plant roots or aerial tissues relative to its concentration in the soil or nutrient solution. BCF was calculated using the formula of Shi et al.^33^.

$$\:\mathrm{BCF}\:=\:\left(\mathrm{Cd}\right)\text{ roots or shoot/}\left(\mathrm{Cd}\right)\text{ soil}$$

### Determination of lipid peroxidation and hydrogen peroxide content

The level of lipid peroxidation was determined by means of the thiobarbituric acid (TBA) assay, which measures the malondialdehyde (MDA) content as a byproduct of the lipid peroxidation reaction. MDA levels were determined spectrophotometrically at 532 nm using an extinction coefficient of 155 mM^−1^ cm^−134^. The hydrogen peroxide content was determined using the method described by Velikova et al.^35^.

### Determination of antioxidant enzyme activity

Fresh shoot samples were extracted for antioxidant enzymes according to de Azevedo Neto et al.^36^. Catalase (CAT) action was assessed in accordance with the method of Beers and Sizer^37^, with some adjustments as explained by a previously reported method^36^. Guaiacol peroxidase (GPX) activity was determined as explained by Urbanek et al.^38^. Enzyme activity was quantified by the quantity of tetraguaiacol produced employing its molar extinction coefficient (26.6 mM^− 1^ cm^− 1^). Superoxide dismutase (SOD) activity was measured based on its ability to inhibit the photochemical reduction of nitroblue tetrazolium chloride (NBT), in accordance with a previously described method^39^.

### Ultra-performance liquid chromatography–triple quadrupole mass spectrometry (UPLC-MS/MS) analysis and metabolite identification of *Allium cepa* L. leaves

UPLC-MS/MS analysis was performed on green leaf samples from treatment groups that showed statistically significant differences in organ dry weight, Cd content, oxidative stress markers, and antioxidant enzyme activities between Cd-stressed and bacterial inoculated Cd-stressed samples, indicative of effective bioremediation. These groups included the untreated control, Cd-stressed samples at 50 and 100 µM, and their corresponding bacterial inoculated counterparts. Samples were collected in triplicate, oven-dried, and extracted with 75% ethanol for three days. The extracts were filtered and concentrated under reduced pressure. A residue of 30 mg from each sample was reconstituted in 3 mL methanol and subsequently filtered through a 0.45 μm syringe filter for UPLC-MS/MS analysis. Analysis was conducted using a UPLC XEVO TQD triple quadrupole mass spectrometer (Waters Corporation, Milford, USA). An 8 µL aliquot of each sample was injected into a Waters Acquity UPLC BEH C18 column (50 mm × 2.1 mm i.d., 1.7 μm particle size), following a previously reported method^40^. Mobile phase consisted of ultrapure water (A) and acetonitrile (B) both acidified with 0.1% formic acid using the following gradient, 0.0–2.0 min, 10% B; 2.0–5.0 min, 30% B; 5.0–15.0 min, 70% B; 22.0 min, 90% B; 22.0–25.0 min, 90% B; 26.0 min, 100% B; 26.0–29.0 min, 100% B; 32.0 min, 10% B. The column temperature was set at 30 °C and flow rate was 0.2 mL/min. UPLC-MS/MS analysis was performed in positive and negative electrospray ionization (ESI) modes, using a capillary voltage of 3 kV, a cone voltage of 30 V, an ion source temperature of 150 °C, and a nebulizer pressure of 35 psi. The temperatures of the drying and sheath gas (nitrogen gas) were 350 °C and 440 °C, respectively, with flow rates of 900 L/h and 50 L/h, respectively. Mass spectra were acquired in full scan mode over the range m/z 100–1250 to enable untargeted metabolite profiling. Automatic MS/MS fragmentation of the precursor ions was performed via collision‑induced dissociation (CID) using an energy ramp from 30 to 70 eV and nitrogen gas as the collision gas.

Mass spectral data were preprocessed using MZmine 2 software (version 2.53, https://github.com/mzmine/mzmine2)^41^, followed by tentative metabolite identification using an untargeted approach to provide a comprehensive profile of the detected metabolites^42^. The method allowed for the detection and annotation of major metabolite classes typically reported in *Allium cepa* L., including amino acids, fatty acids, flavonoids, phenolic acids, and sulfur-containing compounds. Hierarchical clustering (HCA) - heat map analysis of the identified metabolites was performed using Metaboanalyst 6.0 (https://www.metaboanalyst.ca/).

### In vitro α-glucosidase inhibitory effect

In vitro α-glucosidase inhibitory activity of onion green leaves samples was measured according to Pistia-Brueggeman et al. method^43^. In a 96-well microplate, 50 µL of onion leaf extracts (control, Cd-treated, and inoculated Cd-treated) at concentrations ranging from 100 to 1000 µg/mL were added to each well, along with 10 µL of alpha glucosidase enzyme (maltase) (maltase from yeast; Sisco Research Laboratories Pvt. Ltd., 1 U/mL) and 125 µL of 0.1 M phosphate buffer (pH 6.8). The reaction mixtures were preincubated at 37 °C for 20 min. Subsequently, 20 µL of the substrate *p*-nitrophenyl-α-D-glucopyranoside (pNPG) 1 M (Sigma-Aldrich, USA, Cat# N7006) was added to initiate the reaction. The plates were then incubated for an additional 30 min at 37 °C. The reaction was terminated by adding 100 µL of 200 mM sodium carbonate to each well. Absorbance was measured at 405 nm using a microplate reader (Omega, USA). The percentage inhibition of α-glucosidase activity was calculated using the following equation:

$$\% \:\text{of Inhibition} = [(\mathrm{Acontrol}-\mathrm{Asample}\mathrm{)/}\mathrm{Acontrol}\mathrm{]}\text{}\times \mathrm{100}$$

where A_sample_ is the absorbance while the inhibitor is present, and A_control_ is the absorbance of the control (contains phosphate buffer only). Concentration at which 50% inhibition is observed (IC_50_) values were calculated using Graphpad Prism software version 6.07 for Windows (Graph-Pad Software, San Diego, USA).

### Statistical and multivariate data analysis

Experimental data are expressed as the mean ± SD from three independent replicates. One-way analysis of variance (ANOVA) for group comparison was performed on IBM SPSS software version 20.0. (Armonk, NY: IBM Corp), with statistical significance set at *p* < 0.05. Chemometric modeling of the acquired LC–MS data was carried out using SIMCA version 14 (Umetrics, Sweden). An orthogonal projections to latent structures-discriminant analysis (OPLS-DA) model was employed to highlight differences in the chemical profiles of onion samples subjected to cadmium stress in comparison to inoculated and control groups. Orthogonal projection to latent structures (OPLS) model was utilized to identify chemical markers in onion samples correlated to the antidiabetic activity through careful examination of their coefficient plots.

## Results

### Tolerance of bacteria to different concentrations of Cd and prediction of cadmium tolerance genes

*Lb. plantarum* 10CH exhibited tolerance to Cd concentrations up to 100 µM, whereas no colonies were recovered at 200 µM. Quantitative evaluation demonstrated a concentration-dependent reduction in viability, with the 100 µM exposure resulting in a reduction in the mean viable count from 8.5 × 10^8^ to 2.1 × 10^6^ CFU/mL. Furthermore, the WGS annotation of the bacterial genome revealed the presence of several functional genes encoding transport and regulatory systems for cadmium (Table 1; Table S1). The genetic architecture supporting metal resistance includes *cadA*, encoding a P-type ATPase for active Cd^2+^ efflux from the cytoplasm; *czcD* (a member of the cation diffusion facilitator CDF), involved in cobalt/zinc/cadmium homeostasis; multiple P-type ATPases (*copA*, *copB*, *cadC*) with predicted broad substrate specificity (lead, cadmium, zinc, mercury, copper); and *zntA*, encoding an accessory protein for Cd efflux. Collectively, these loci suggest a coordinated, broad-spectrum heavy metal resistance mechanism. Together, these genes indicate that *Lb. plantarum* 10CH possesses a set of efflux-based resistance determinants consistent with an adaptive strategy for maintaining cytoplasmic metal homeostasis under cadmium stress.

**Table 1:** Heavy metal tolerance genes identified in *Lb. plantarum* 10CH genome sequence via BV-BRC platform.

Gene	Product
*cadA*	Cadmium-transporting ATPase (EC 3.6.3.3)
*czcD*	Cobalt/zinc/cadmium resistance protein CzcD
*copA*	Lead, cadmium, zinc and mercury transporting ATPase; Copper-translocating P-type ATPase
*copB*	Lead, cadmium, zinc and mercury transporting ATPase; Copper-translocating P-type ATPase
*cadC*	Lead, cadmium, zinc and mercury transporting ATPase; Copper-translocating P-type ATPase
*zntA*	Cadmium efflux system accessory protein

### Effect of *Lb. plantarum* 10CH on root and shoot dry weight of *Allium cepa* L. following Cd treatment

**Fig. 1:**
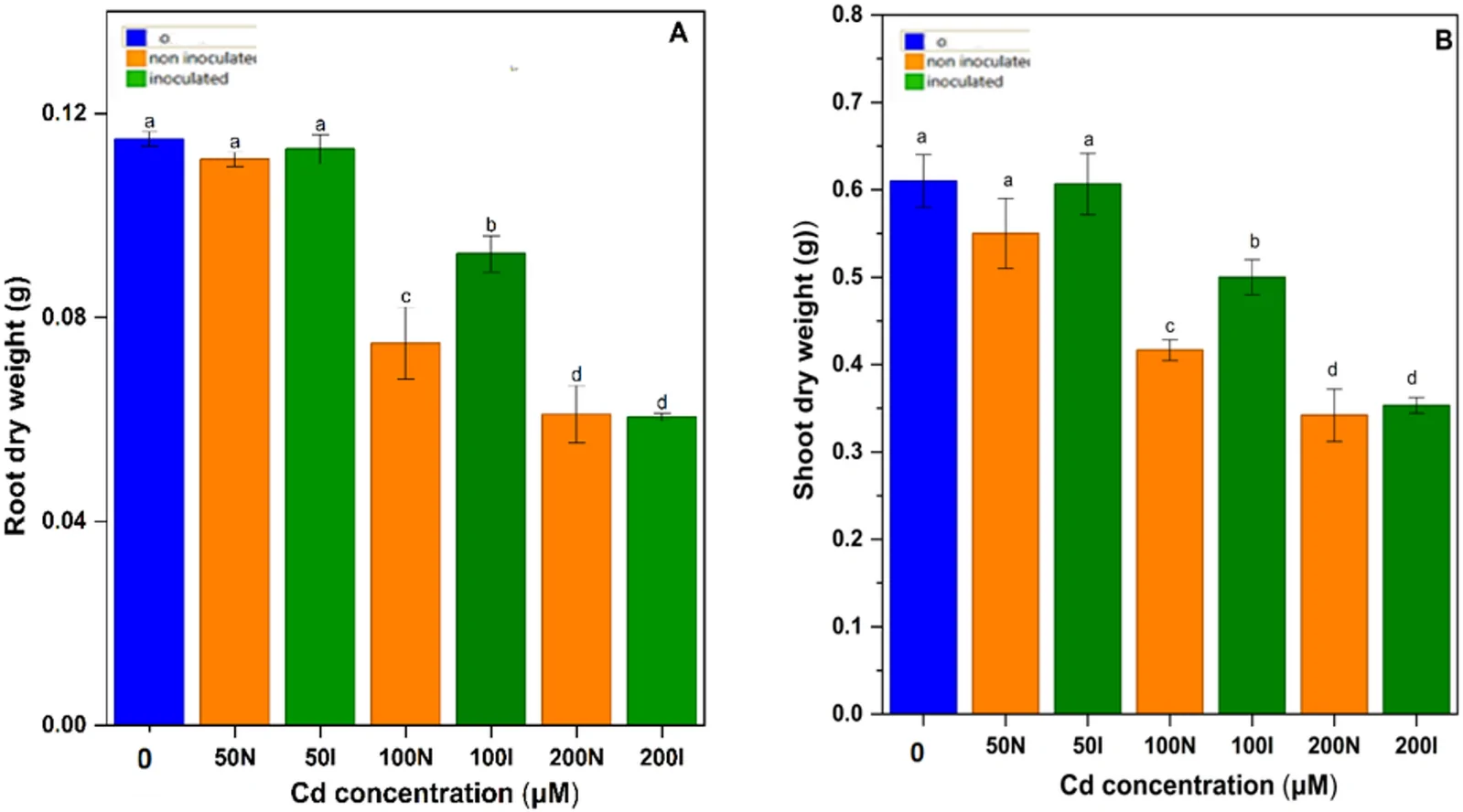
Effect of *Lb. plantarum* 10CH on (**A**) root dry weight and (**B**) shoot dry weight of *Allium cepa* L. following treatment with different concentrations of CdCl_2_. 0 = control untreated; 50 *N* = 50 µM Cd without bacterial inoculation; 50I = 50 µM Cd with bacterial inoculation; 100 *N* = 100 µM Cd without inoculation; 100I = 100 µM Cd with bacterial inoculation; 200 *N* = 200 µM Cd without inoculation; and 200I = 200 µM Cd with bacterial inoculation. Data are mean ± SD of triplicates. Different letters indicate statistically significant at *p* < 0.05 as evaluated by Tukey’s HSD post-hoc.

Exposure to higher concentrations of CdCl_2_ resulted in a significant reduction in root and shoot biomass. At 100 µM CdCl_2_, root and shoot biomass decreased by 35% and 32%, respectively, whereas at 200 µM, the reductions reached 47% and 42%, respectively. At a lower concentration of 50 µM, cadmium did not show a significant effect on organs biomass. However, bacterial inoculation with *Lb. plantarum* 10CH mitigated the negative impact of 100 µM Cd on root and shoot biomass, showing improvements of 23% and 20%, respectively (Fig. 1). At a cadmium treatment of 200 µM, bacterial inoculation did not confer any significant improvement in plant growth.

### Effect of *Lb. plantarum* 10CH on Cd content and Cd BCF of *Allium cepa* L. following Cd treatment

**Fig. 2:**
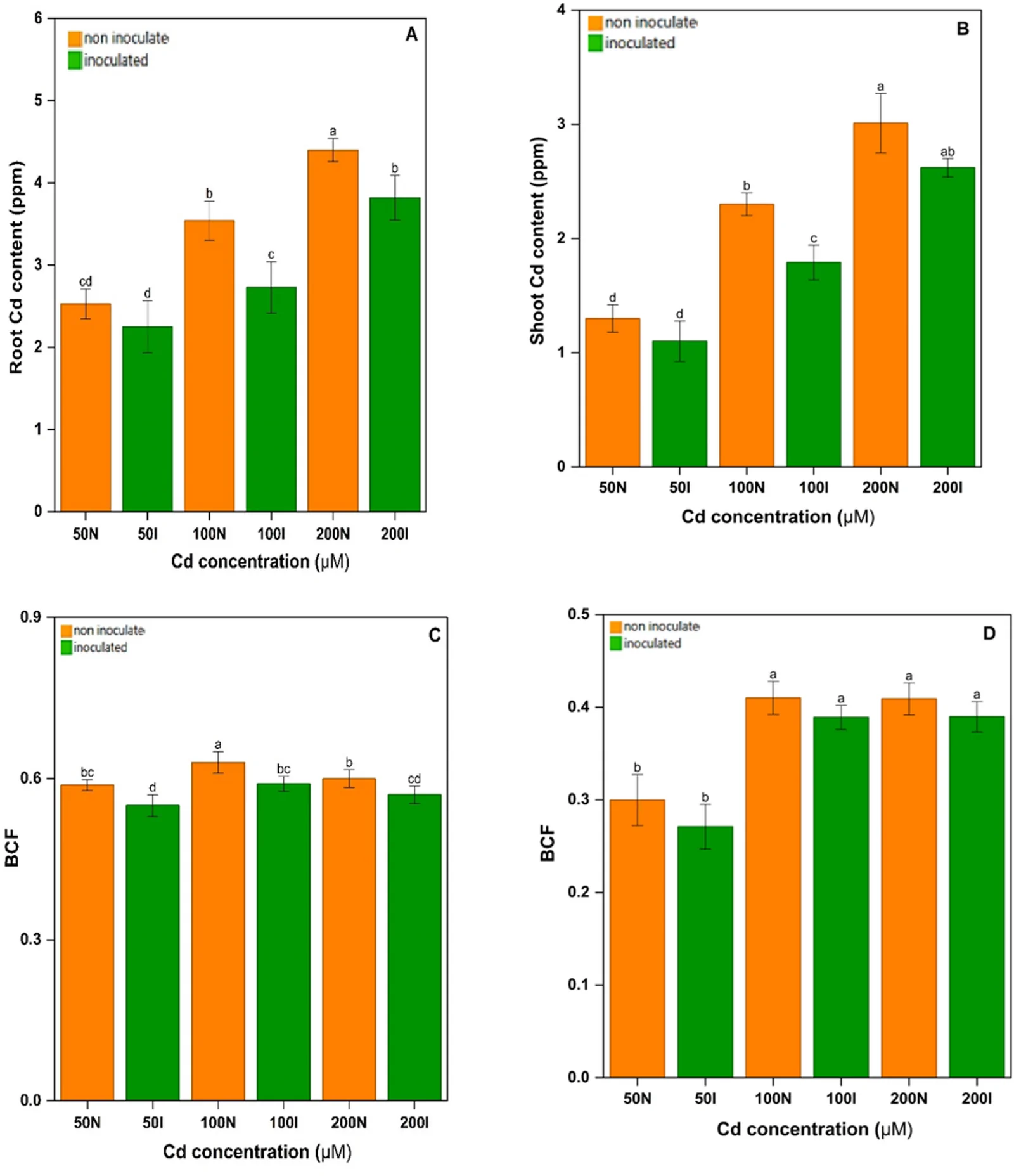
Effect of *Lb. plantarum* 10CH on Cd content in (**A**) root, (**B**) shoot, and on the BCF in (**C**) root and (**D**) shoot of *Allium cepa* L. following treatment with different concentrations of CdCl_2_. 50 *N* = 50 µM Cd without bacterial inoculation; 50I = 50 µM Cd with bacterial inoculation; 100 *N* = 100 µM Cd without inoculation; 100I = 100 µM Cd with bacterial inoculation; 200 *N* = 200 µM Cd without inoculation; and 200I = 200 µM Cd with bacterial inoculation. Data are mean ± SD of triplicates. Different letters indicate statistically significant at *p* < 0.05 as evaluated by Tukey’s HSD post-hoc.

Cd concentration and BCF in the roots and shoots are illustrated in Fig. 2. Overall, Cd accumulation was higher in roots than in shoots, as indicated by the elevated Cd concentrations and BCF values in root samples. Bacterial inoculation significantly reduced Cd accumulation by 23% and 22% in roots and shoots, respectively, at 100 µM. However, bacterial inoculation significantly lowered Cd levels at 200 µM Cd in root samples by 13%, but no significant change was observed in shoots.

### Effect of *Lb. plantarum* 10CH on lipid peroxidation and hydrogen peroxide content of *Allium cepa* L. shoots following Cd treatment

**Fig. 3:**
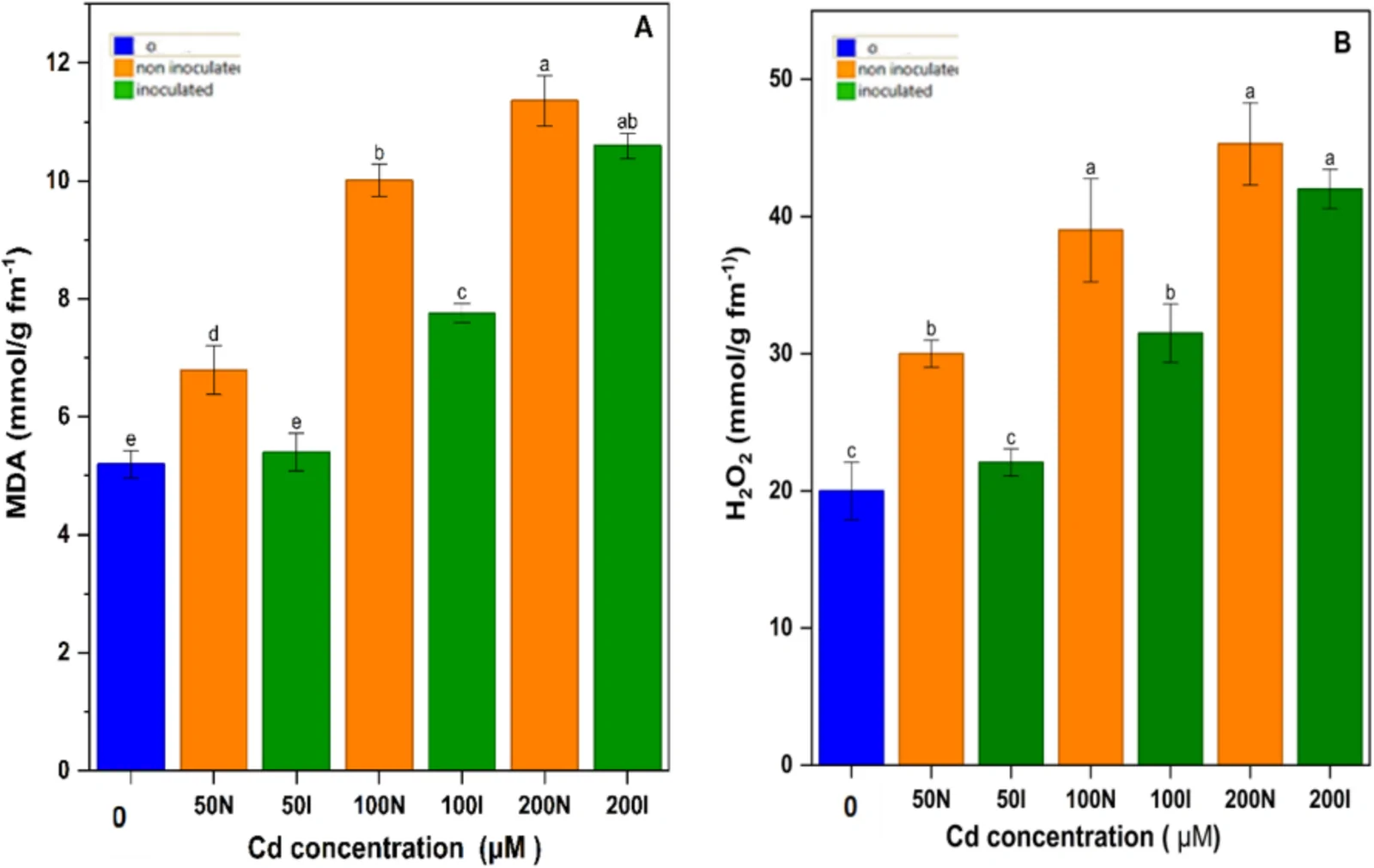
Effect of *Lb. plantarum* 10CH on (**A**) lipid peroxidation and (**B**) hydrogen peroxide content of *Allium cepa* L. shoots following treatment with different concentrations of CdCl_2_. 0 = control untreated ; 50 *N* = 50 µM Cd without bacterial inoculation; 50I = 50 µM Cd with bacterial inoculation; 100 *N* = 100 µM Cd without inoculation; 100I = 100 µM Cd with bacterial inoculation; 200 *N* = 200 µM Cd without inoculation; and 200I = 200 µM Cd with bacterial inoculation. Data are mean ± SD of triplicates. Different letters indicate statistically significant at *p* < 0.05 as evaluated by Tukey’s HSD post-hoc.

The MDA and H_2_O_2_ contents of non-inoculated plants increased significantly with increasing CdCl_2_ concentration compared with the untreated plants (Fig. 3). However, bacterial inoculation significantly decreased their levels, as inoculated plants exposed to 100 µM CdCl_2_ showed reductions in MDA and H_2_O_2_ by 28% and 24%, respectively.

### Effect of *Lb. plantarum* 10CH on the antioxidant activity of *Allium cepa* L. shoots following Cd treatment

**Fig. 4:**
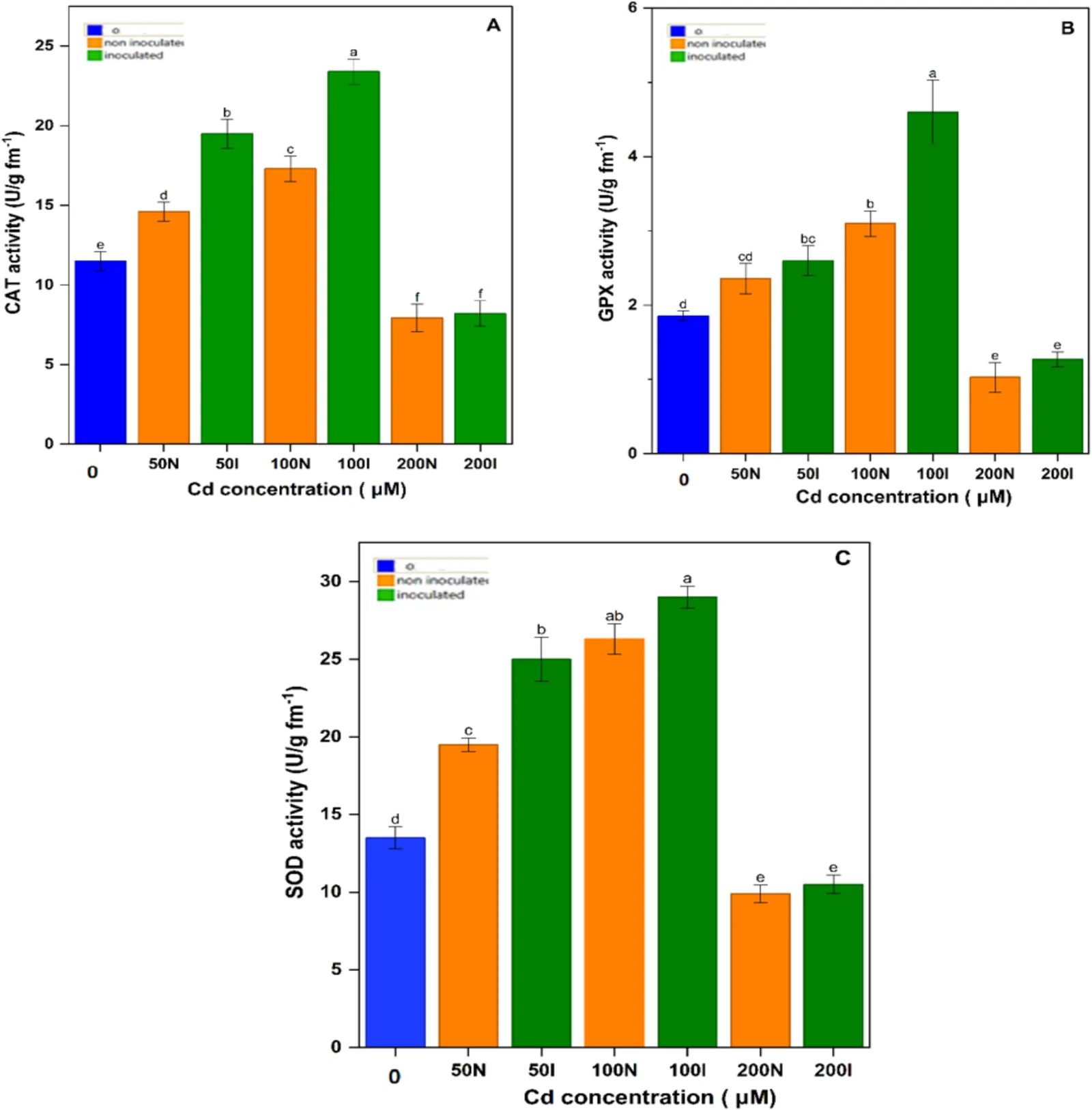
Effect of *Lb. plantarum* 10CH on the activity of antioxidant enzymes of *Allium cepa* L. shoots following treatment with different concentrations of CdCl_2_. (**A**) catalase (CAT), (**B**) guaiacol peroxidase (GPX), (**C**) superoxide dismutase enzyme (SOD). 50 *N* = 50 µM Cd without bacterial inoculation; 0 = control untreated; 50I = 50 µM Cd with bacterial inoculation; 100 *N* = 100 µM Cd without inoculation; 100I = 100 µM Cd with bacterial inoculation; 200 *N* = 200 µM Cd without inoculation; and 200I = 200 µM Cd with bacterial inoculation. Data are mean ± SD of triplicates. Different letters indicate statistically significant at *p* < 0.05 as evaluated by Tukey’s HSD post-hoc.

Cadmium stress in onion shoots at 50 and 100 µM CdCl_2_ concentrations resulted in a significant increase in the activities of the antioxidant enzymes catalase (CAT), guaiacol peroxidase (GPX), and superoxide dismutase (SOD) compared with untreated plants. In contrast, exposure to 200 µM CdCl_2_ led to a decline in the activities of these enzymes (Fig. 4). Bacterial inoculation significantly enhanced CAT and GPX activities in plants exposed to 100 µM CdCl_2_ by 26% and 32%, respectively, while SOD activity showed a 9% increase. Inoculated plants treated with 50 µM CdCl_2_ also exhibited increased CAT and SOD levels, whereas bacterial inoculation at 200 µM CdCl_2_ did not cause any significant changes in antioxidant enzyme levels (Fig. 4).

### UPLC-MS/MS chemical profiling and OPLS-DA analysis of *Allium cepa* L. leaves

Comparison of organ dry weight, Cd content, oxidative stress markers (lipid peroxidation and hydrogen peroxide content), and antioxidant enzyme activities between Cd-treated and *Lb. plantarum* 10CH-inoculated Cd-treated onion plants showed that, although bacterial inoculation significantly reduced root Cd content by 13% at 200 µM, this concentration did not produce significant amelioration of Cd-induced effects in leaf tissue (Fig. 2). Metabolic profiling was therefore conducted on leaves from plants stressed at 50 and 100 µM Cd, where bacterial inoculation resulted in significant reduction in Cd content and enhancement of antioxidant enzyme activities (Figs. 1, 2, 3 and 4). UPLC-MS/MS was accordingly employed to assess the impact of Cd stress on the phytochemical composition of *A. cepa* L. leaves compared to the control and to evaluate chemical profile alterations upon *Lb. plantarum* 10CH inoculation of Cd-treated samples.

As shown in Figure S1 and Table 2, a total of 80 metabolites were identified through comparison of their retention times and fragmentation patterns with external standards, literature data^25,27,44,45^, and our in-house database for natural compounds previously reported in *A. cepa* L. Amino acids and peptides represented the most abundant class (25 compounds), followed by 21 flavonoids. Together, these two classes accounted for nearly half of the annotated metabolites (Table 2). Among the amino acids and peptides, several sulfur-containing compounds, such as methionine S-oxide, allicin, methionine, and others, were detected. The detected flavonoids belonged to four main subclasses: flavonols, such as quercetin, kaempferol, and their glycosides; flavanones (naringenin, hesperetin); isoflavones, such as biochanin A; and anthocyanin glycosides. Other classes included saponins, phenolic acids, and fatty acids (Table 2).

**Fig. 5:**
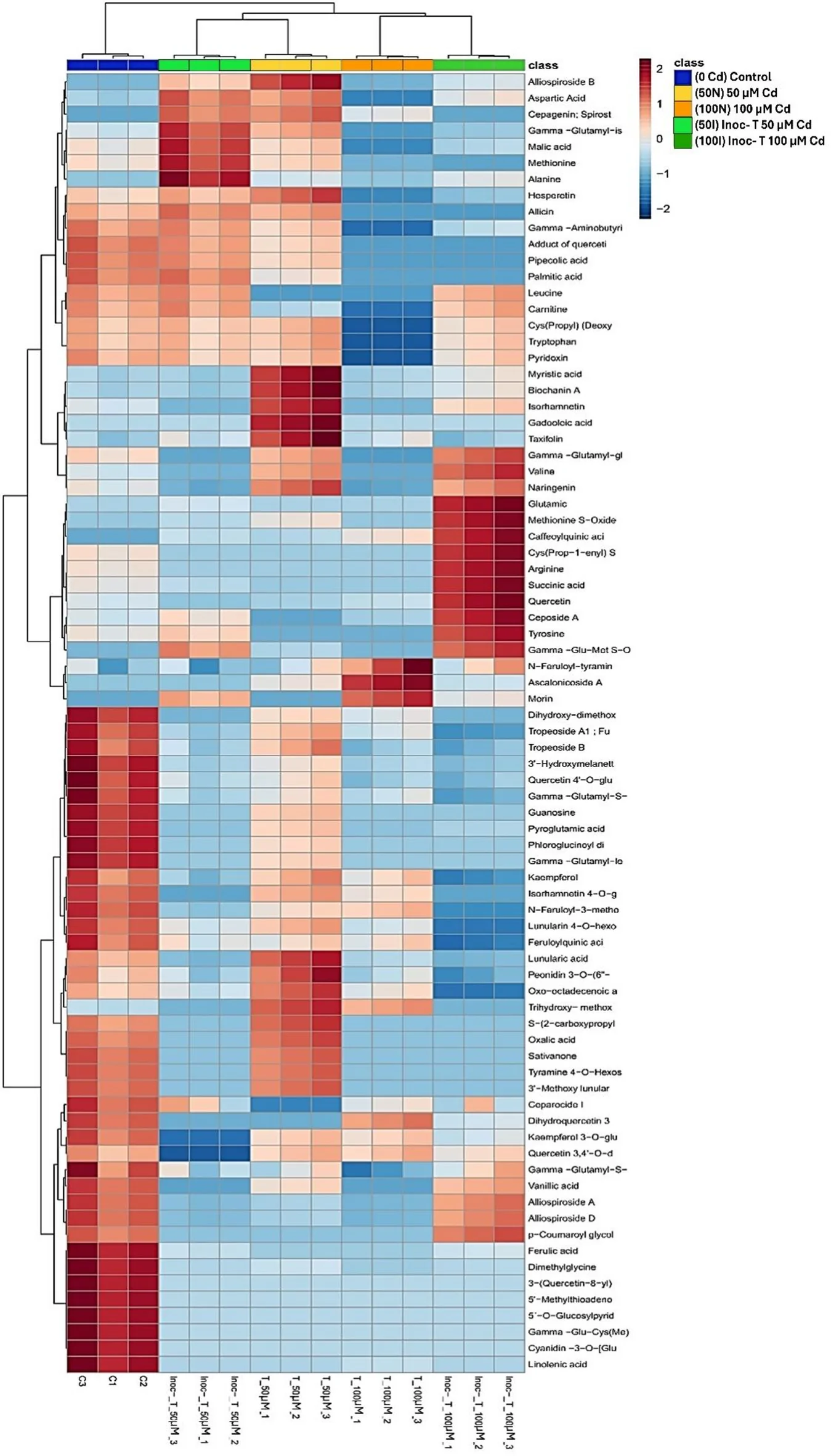
HCA dendrogram-heat map of relative distribution of identified phytoconstituents of *Allium cepa* L. leaves samples. Color gradient from red to blue indicates relative distribution (from highest to lowest).

The relative distribution of the identified metabolites in onion leaf samples is illustrated in a heat map in Fig. 5. Hierarchical clustering analysis (HCA) revealed a clear separation between control samples and those treated with Cd, with or without bacterial inoculation, indicating that Cd exposure significantly alters metabolite accumulation patterns. Compared to the control, treatment with 100 µM Cd induced pronounced alterations in the primary metabolites, notably marked by the depletion of most amino acids and peptides. Meanwhile, flavonoids mainly of the flavonol type, such as quercetin 3,4’-O-diglucoside, kaempferol 3-O-glucoside, kaempferol, and morin, were detected. Additionally, three saponins: cepagenin, ceparocide I, and ascalonicoside A were detected, with ascalonicoside A being the most abundant in the 100 µM Cd-treated samples. Moreover, N-feruloyl-tyramine, a hydroxycinnamic acid amide, was also prominently accumulated in these samples (Fig. 5).

However, treatment with 50 µM Cd had a minor effect on the primary metabolite production, where nearly most amino acids, peptides, and fatty acids were detected. Regarding secondary metabolites, the 50 µM Cd-treated samples were characterized by the presence of flavonoid genins (taxifolin, biochanin A, and isorhamnetin), fatty acids (myristic and gadoleic acids), and the saponin alliospiroside B as dominant compounds.

Bacterial inoculation of the 100 µM Cd-treated plants appeared to mitigate Cd-induced suppression of primary metabolites, restoring the levels of several amino acids and peptides. Notably, arginine, tyrosine, methionine S-oxide, γ-Glu-Met S-oxide, isoalliin, and glutamic acid showed markedly higher abundance following bacterial inoculation. Similarly, in the 50 µM Cd-treated samples, bacterial inoculation enhanced the levels of amino acids such as alanine and methionine, as well as peptides including γ-glutamyl-isoleucine and γ-Glu-Met S-oxide. However, inoculation at both Cd concentrations exerted minimal effects on the accumulation of flavonoids, saponins, and other secondary metabolites (Fig. 5).

**Table 2:** The identified metabolites in the ethanolic extracts of *Allium cepa* L. leaves samples (control, Cd-treated, and inoculated with *Lb. plantarum* 10CH following Cd stress) analyzed using UPLC-MS/MS.

No	RT (min)	Mass (m/z)	Compound name	Formula	Class	MS^*n*^ ions m/z	Ion Type	Reference
1	0.407	113.91	Oxalic acid	C_2_H_2_O_4_	Organic acid	91, 73	[M + Na]^+^	^45^
2	0.633	131.99	Carnitine	C_7_H_16_NO⁺	Amino acid	162, 103, 102, 60	[M + H]^+^	^44^
3	0.758	131.98	Alanine	C_3_H_7_NO_2_	Amino acid	44, 45	[M+aceto + H]^+^	^46^
4	0.769	104.04	Gamma -Aminobutyric acid	C_4_H_9_NO_2_	Gamma amino acids	104	[M + H]^+^	^44^
5	0.812	175.08	Arginine	C_6_H_14_N_4_O_2_	Amino acid	175	[M + H]^+^	^44^
6	0.844	175.08	Aspartic Acid	C_4_H_7_NO_4_	Amino acid	134	[M+aceto + H]^+^	^44^
7	0.916	185	Methionine S-Oxide	C_5_H_11_NO_3_S	Amino acid	166	[M + H+H_2_O]^+^	^44^
8	1.058	146.86	Glutamic	C_5_H_9_NO_4_	Amino acid	128, 102	[M-H]^−^	^44^
9	1.079	162.88	Valine	C_5_H_11_NO_2_	Amino acid	98, 74, 72, 44	[M-H+HCOO]^−^	^44^
10	1.091	314.12	Gamma -Glutamyl-glutamine	C_10_H_17_N_3_O_6_	Peptide	147, 130, 101, 84	[M + K]^+^	^44^
11	1.114	179.06	Allicin	C_6_H_10_OS_2_	Thiosulfinic acid esters	120, 89,48	[M-H+H2O]^−^	^45^
12	1.1	298.08	Gamma -Glu-Cys(Me) S-Oxide	C_9_H_16_N_2_O_6_S	N-acyl-amino acid	217, 154, 130, 88	[M+NH_4_]^+^	^44^
13	1.192	148.99	Gamma -Glu-Met S-Oxide	C_10_H_18_N_2_O_6_S	Peptide	277,166, 130	[M/2 + H]^+^	^44^
14	1.211	117.98	Malic acid	C_4_H_6_O_5_	Organic acid	117, 99,75	[M + H-H_2_O]^+^	^46^
15	1.2108	182.08	Tyrosine	C_9_H_11_NO_3_	Amino acid	165, 147,136,123,91	[M + H]^+^	^46,47^
16	1.214	217.08	Cys(Prop-1-enyl) S-Oxide (Isoalliin)	C_6_H_11_NO_3_S	Amino acid	161,137,120	[M + K]^+^	^44^
17	1.248	164.1	Pipecolic acid	C_6_H_11_NO_2_	Amino acid	128, 82	[M + Cl]^−^	^44^
18	1.523	297.23	Methionine	C_5_H_11_NO_2_S	Amino acid	115, 100, 47	[2 M-H]^−^	^44^
19	1.777	148.96	Pyroglutamic acid ; (pyro-Glu)	C_5_H_7_NO_3_	Amino acid	84, 56, 41	[M + H+H_2_O]+	^45^
20	1.523	205.11	p-Coumaroyl glycolic acid	C_11_H_10_O_5_	Phenolic acid	205, 147, 119	[M+aceto + H]+	^45^
21	1.951	205.09	Tryptophan	C_11_H_12_N_2_O_2_	Amino acid	188, 146, 144, 118, 91	[M + H]^+^	^45^
22	1.971	203.13	Vanillic acid	C_8_H_8_O_4_	Phenolic acid	153,137, 123	[M + Cl]^−^	^45^
23	1.972	119.98	Succinic acid	C_4_H_6_O_4_	Organic acid	118.09	[M + H]^+^	^45^
24	2.007	188.04	Pyridoxin	C_8_H_11_NO_3_	Pyridoxines	152, 134, 124	[M + H+H_2_O]^+^	^44^
25	2.016	104.03	Dimethylglycine	C_4_H_9_NO_2_	Amino acid	60, 44	[M + H]^+^	^46^
26	2.315	**132**	Leucine	C_6_H_13_NO_2_	Amino acid	86, 69, 44	[M + H]^+^	^44^
27	2.428	104.02	S-(2-carboxypropyl)cysteine	C_7_H_13_NO_4_S	Cysteine derivatives	191, 119	[M/2 + H]^+^	^44^
28	2.541	131.97	Gamma -Glutamyl-isoleucine	C_11_H_20_N_2_O_5_	Peptide	215, 169,132, 86	[M/2 + H]^+^	^44^
29	2.844	131.99	Gamma -Glutamyl-leucine	C_11_H_20_N_2_O_5_	Peptide	244, 198,130	[M/2 + H]^+^	^45^
30	3.508	329.29	Gamma -Glutamyl-S-(Propyl) cysteine sulfoxide	C_11_H_20_N_2_O_6_S	Sulfur-containing peptide	215, 178, 152, 128	[M-2 H + Na]^−^	^44^
31	4.414	205.09	Cys(Propyl); (Deoxypropiin)	C_6_H_13_NO_2_S	Cysteine derivatives	147, 105, 87	[M+aceto + H]^+^	^44^
32	5.003	197.11	Gamma -Glutamyl-S-(2-carboxypropyl) cysteine-glycine	C_14_H_23_N_3_O_8_S	Sulfur-containing peptide	263,87,74	[M/2 + H] ^+^	^45^
33	5.401	625.29	Quercetin 3,4’-O-diglucoside	C_27_H_30_O_17_	Flavonoid	463, 445, 301, 179, 161	[M-H]^−^	^45^
34	5.697	901.68	Cyanidin − 3-O-[Glucopyranosyl-(1→3)-6-O-malonyl-glucopyranoside], 4’-O-glucopyranoside	C_36_H_43_O_24_	Flavonoid	739, 653, 577	[M + K]^+^	^45^
35	6.176	463.16	Quercetin 4’-O-glucoside	C_21_H_20_O_12_	Flavonoid	301, 179, 151	[M-H]^−^	^45^
36	6.484	917.56	Ascalonicoside A	C_45_H_74_O_19_	Saponin	771, 609, 447, 161	[M-H]^−^	^45^
37	6.548	447.15	Kaempferol 3-O-glucoside	C_21_H_20_O_11_	Flavonoid	285, 284, 255, 151	[M-H]^−^	^44^
38	6.628	477.19	Isorhamnetin 4-O-glucoside	C_22_H_22_O_12_	Flavonoid	315, 299, 151, 137	[M-H]^−^	^46^
39	6.75	871.62	Ceposide A	C_44_H_72_O_18_	Saponin	743, 611,581,449	[M + H-H_2_O]^+^	^45^
40	6.787	887.7	Dihydroquercetin 3-O-rhamnoside	C_21_H_22_O_11_	Flavonoid	301, 283, 149, 107	[M-H]^−^	^44^
41	6.799	871.63	Ceparocide I	C_44_H_70_O_17_	Saponins	72, 593, 575	[M + H]^+^	^45^
42	6.85	166.07	5’-O-Glucosylpyridoxin	C_14_H_21_NO_8_	Pyridoxine glycoside	314, 152, 136, 108	[M/2 + H]^+^	^44^
43	6.989	318.31	Tyramine 4-O-Hexoside	C_14_H_21_NO_6_	Phenolic glycoside	138, 121	[M + H-H_2_O]^+^	^44^
44	7.322	284.12	Guanosine	C_10_H_13_N_5_O_5_	Purine nucleosides	152, 135	[M + H]^+^	^44,48^
45	7.478	314.13	N-Feruloyl-tyramine	C_18_H_19_NO_4_	Hydroxycinnamic acid amide	177, 138, 121	[M + H]^+^	^45^
46	7.607	344.16	N-Feruloyl-3-methoxytyramine	C_19_H_21_NO_5_	Hydroxycinnamic acid amide	177, 168	[M + H]^+^	^45^
47	7.631	342.2	5’-Methylthioadenosine	C_11_H_15_N_5_O_3_S	5’-deoxy-5’-thionucleosides	134	[M-H+HCOO]^−^	^44^
48	8.798	275.17	Peonidin 3-O-(6’’-O-Malonyl-glucopyranoside)	C_25_H_25_O_14_	Flavonoid	463, 301	[M/2 + H]^+^	^49^
49	8.798	351.23	Feruloylquinic acid	C_17_H_20_O_9_	Phenolic acid	195,193, 177	[M + H-H_2_O]^+^	^45^
50	8.917	395.29	Lunularin 4-O-hexoside	C_20_H_24_O_7_	Stilbenoid glycoside	271, 215	[M + H+H_2_O]^+^	^45^
51	9.37	372.24	Caffeoylquinic acid	C_16_H_18_O_9_	Phenolic acid	181,175, 163, 161, 135	[M+NH4]^+^	^45^
52	9.677	623.42	3-(Quercetin-8-yl)-2,3-epoxyflavanone	C_30_H_18_O_14_	Flavonoid	449, 299	[M-2 H + Na]^−^	^45^
53	9.721	217.08	Ferulic acid	C_10_H_10_O_4_	Phenolic acid	180, 151, 136	[M + Na]^+^	^45^
54	10.366	280.25	Phloroglucinoyl dihydroxybenzoate	C_13_H_10_O_6_	Dihydroxybenzoic acid derivative	155, 127, 111	[M+NH4]^+^	^45^
55	11.915	739.52	Tropeoside A1 ; Furost-5-ene-1,3,22,26-tetrol; 1-O-Galactopyranoside, 3-O-rhamnopyranoside	C_39_H_64_O_14_	Saponin	577, 431	[M + H-H2O]^+^	^50^
56	11.98	753.57	Alliospiroside D	C_39_H_62_O_14_	Saponin	607, 445,427,515	[M-H]^−^	^46^
57	12.487	783.6	Alliospiroside B	C_39_H_62_O_13_	Saponin	593, 431, 413, 397	[M + H]^+^	^45^
58	12.981	709.5	Tropeoside B	C_38_H_62_O_13_	Saponin	595, 449, 430	[M + H-H_2_O]^+^	^45^
59	13.061	753.57	Alliospiroside A	C_38_H_60_O_12_	Saponin	561, 429, 411, 395	[M-H+HCOO]^−^	^45^
60	15.548	325.14	Quercetin	C_15_H_10_O_7_	Flavonoid	273, 181, 153	[M + Na]^+^	^45^
61	15.756	344.33	Morin	C_15_H_10_O_7_	Flavonoid	273, 153, 139	[M+aceto + H]^+^	^45^
62	15.813	496.37	Adduct of quercetin with protocatechuic acid	C_22_H_14_O_11_	Flavonoid	439, 303	[M+aceto + H]^+^	^45^
63	15.977	322.27	Taxifolin	C_15_H_12_O_7_	Flavonoid	287, 277, 243, 181, 153	[M+NH_4_]^+^	^45^
64	16.019	353.25	Isorhamnetin	C_16_H_12_O_7_	Flavonoid	273, 257 ,151, 135	[M-2 H + K]^−^	^45^
65	16.163	277.18	Lunularic acid	C_15_H_14_O_4_	Phenolic acid	215, 109, 108	[M + H-H2O]^+^	^44,45^
66	16.682	285.04	Kaempferol	C_15_H_10_O_6_	Flavonoid	243, 151, 133	[M-H]^−^	^44^
67	16.826	339.28	Trihydroxy- methoxyisoflavanone	C_16_H_14_O_6_	Flavonoid	231, 151, 135	[M-2 H + K]^−^	^45^
68	17.097	339.27	Hesperetin	C_16_H_14_O_6_	Flavonoid	164, 151, 136	[M-2 H + K]^−^	^45^
69	17.161	318.28	3’-Hydroxymelanettin	C_16_H_12_O_6_	Flavonoid	273, 255, 241, 227	[M+NH_4_]^+^	^45^
70	17.308	293.24	Naringenin	C_15_H_12_O_5_	Flavonoid	150, 118, 106	[M-2 H + Na]^−^	^45^
71	17.916	325.25	3’-Methoxy lunularic acid	C_16_H_16_O_5_	Stilbenoid (phenolic acid)	243, 228, 150, 136	[M-2 H + K]^−^	^44^
72	18.063	353.29	Dihydroxy-dimethoxyisoflavone	C_17_H_14_O_6_	Flavonoid	287, 271, 163	[M + K]^+^	^45^
73	18.08	265.2	Biochanin A	C_16_H_12_O_5_	Flavonoid	239, 227, 201, 185	[M-H-H_2_O]^−^	^44^
74	18.34	318.31	Sativanone	C_17_H_16_O_5_	Flavonoid	273, 165, 137, 121	[M+NH_4_]^+^	^45^
75	18.826	447.41	Cepagenin; Spirost-5-ene-1,3,24-triol	C_27_H_42_O_5_	Saponin	429, 411, 359, 287	[M + H]^+^	^51^
76	19.432	279.2	Oxo-octadecenoic acid	C_18_H_32_O_3_	Fatty acid	279, 261, 147	[M + H-H_2_O]^+^	^45^
77	19.591	277.26	Linolenic acid	C_18_H_30_O_2_	Fatty acid	259, 233, 221, 131	[M-H]^−^	^45^
78	21.034	455.43	Myristic acid	C_14_H_28_O_2_	Fatty acid	209, 183, 71	[2 M-H]^−^	^45^
79	21.618	279.22	Palmitic acid	C_16_H_32_O_2_	Fatty acid	239, 221, 69	[M + Na]^+^	^45^
80	23.075	293.26	Gadooleic acid	C_20_H_38_O_2_	Fatty acid	293, 275, 265, 109	[M + H-H_2_O]^+^	^45^

Multivariate data analysis using the unsupervised PCA model (Figure S2) revealed a clear distinction among the control, Cd-treated, and *Lb. plantarum* 10CH inoculated samples, accounting for 42% of the variance along PC1 and 22% along PC2. This was further confirmed by the supervised (OPLS-DA) model, indicating the alterations in the chemical composition of the investigated onion leaf samples.

**Fig. 6:**
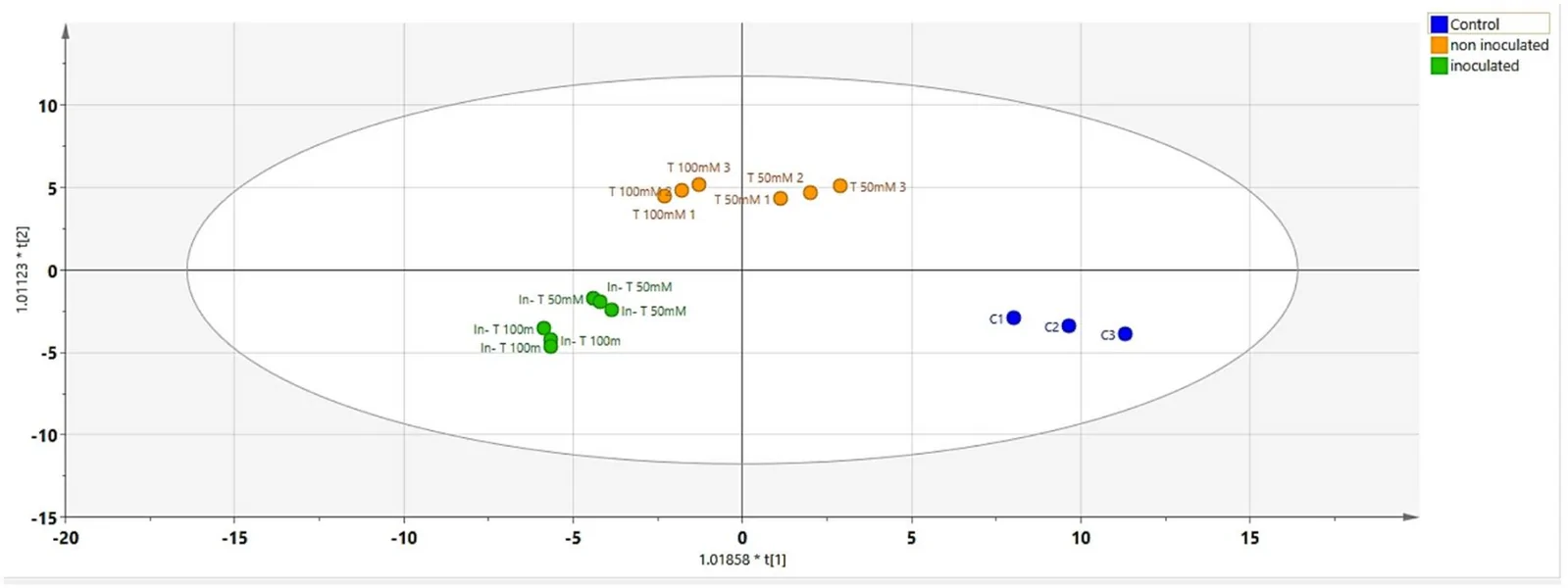
OPLS-DA score plot of *Allium cepa* L. leaves samples.

The constructed OPLS-DA model showed a strong fit with an R^2^Y value of 0.994 and a satisfactory Q^2^ value of 0.899, indicating high reliability and good predictive performance. As shown in the scatter plot (Fig. 6), the control samples and 50 µM Cd-treated samples were clustered along the positive side of latent variable 1 (LV1), whereas 100 µM Cd-treated samples and the inoculated Cd-treated samples at both concentrations were positioned on the negative side of LV1. Further separation was achieved along latent variable 2 (LV2), where control samples and inoculated Cd-treated samples were clustered on the negative side of LV2. In contrast, both 50 µM and 100 µM Cd-treated samples were distributed on the positive side of LV2.

**Fig. 7:**
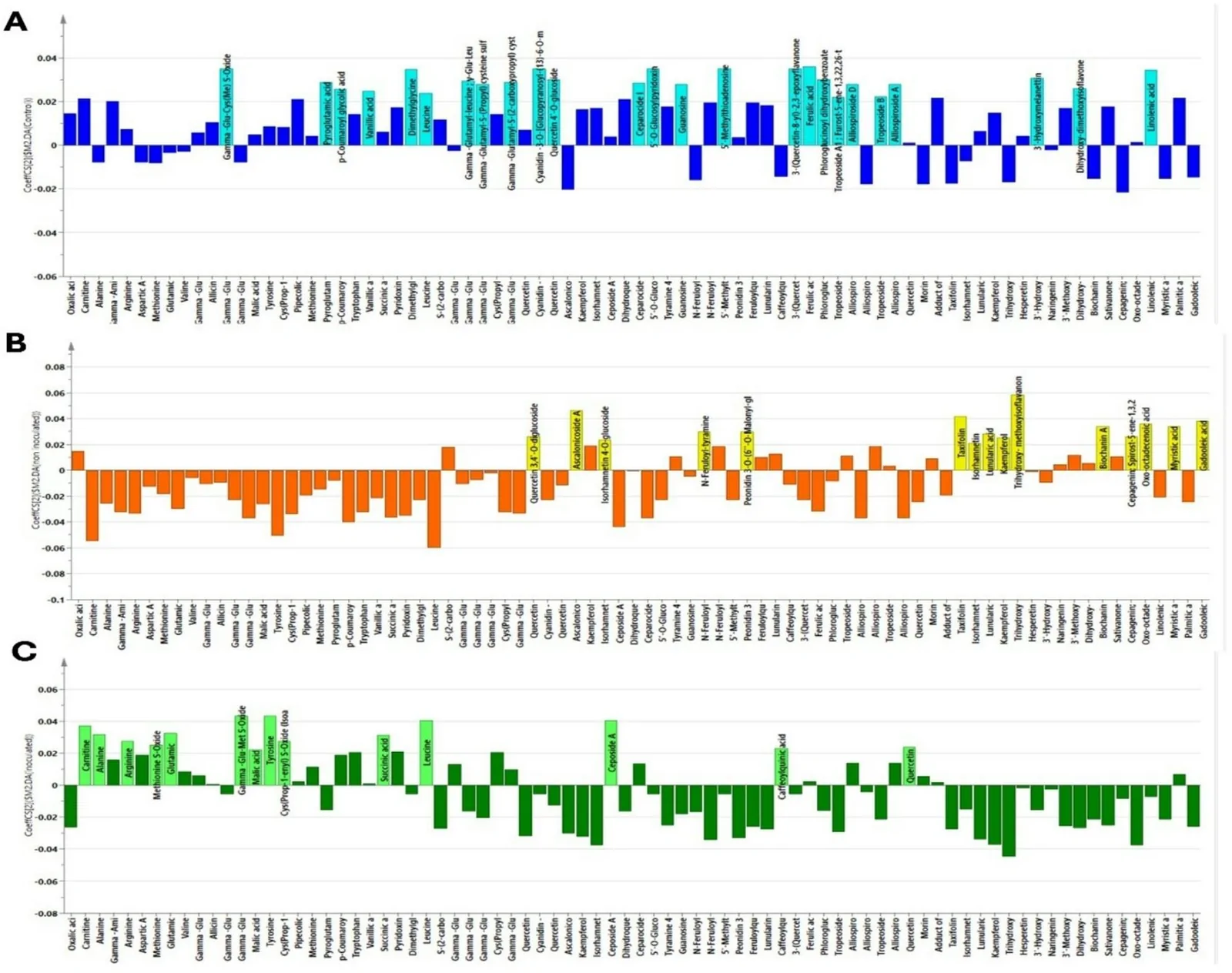
Coefficient plots of constructed OPLS-DA model of *Allium cepa* L. leaves control samples (**A**), Cd-treated (**B**), and *Lb. plantarum* 10CH inoculated Cd-treated (**C**).

Investigating the coefficient plots (Fig. 7A–C) provided the chemical markers distinguishing the control, Cd-treated, and inoculated Cd-treated onion leaves. The control samples were characterized by a wide array of metabolites (Fig. 7A), notably flavonoids such as cyanidin-3-O-[glucopyranosyl-(1→3)-6-O-malonyl-glucopyranoside], quercetin 4′-O-glucopyranoside, 3-(quercetin-8-yl)-2,3-epoxyflavanone, 3′-hydroxymelanettin, and a range of amino acids and peptides including dimethylglycine, γ-glutamyl-leucine, and γ-Glu-Cys(Me) S-oxide. Other discriminating markers included ferulic acid, phloroglucinoyl dihydroxybenzoate, 5′-O-glucosylpyridoxin, 5’-methylthioadenosine, and linolenic acid.

In contrast, Cd-treated samples exhibited positive discriminant markers, including flavonoids (trihydroxy-methoxyisoflavanone, taxifolin, biochanin A, peonidin 3-O-(6″-O-malonyl-glucopyranoside), quercetin 3,4′-diglucoside, kaempferol, isorhamnetin, isorhamnetin 4-O-glucoside), saponins (ascalonicoside A, cepagenin), fatty acids (myristic, gadoleic, oxo-octadecenoic acids), and phenolic compounds (N-feruloyl-tyramine, lunularic acid).

Bacterial inoculation notably influenced the metabolite profile, as evidenced in the heat map (Fig. 5) and further supported by the coefficient plots. A shift in marker compounds was observed, with increased abundance of amino acids and peptides such as tyrosine, leucine, alanine, glutamic acid, arginine, carnitine, methionine S-oxide, γ-Glu-Met S-oxide, and isoalliin. Besides, the saponin ceposide A, flavonoid quercetin, and organic acids like succinic acid and caffeoylquinic acid were prominent discriminators of the inoculated Cd-treated samples.

Direct comparison of Cd-stressed and inoculated Cd-stressed samples showed that bacterial inoculation shifted the levels of several metabolite classes toward those of the untreated control, predominantly primary metabolites (Figs. 5 and 7). Amino acids represented the major affected class (14 compounds), including arginine, aspartic acid, carnitine, Cys(propyl) (deoxypropiin), glutamic acid, gamma-aminobutyric acid, isoalliin, leucine, methionine, methionine S-oxide, pipecolic acid, tryptophan, tyrosine, and valine. Four peptides were also affected: gamma-glutamyl-glutamine, gamma-glutamyl-isoleucine, gamma-glutamyl-methionine S-oxide, and gamma-glutamyl-S-(2-carboxypropyl)cysteine, along with one fatty acid (palmitic acid), the organic acids malic acid and succinic acid, the vitamin pyridoxine, and the organosulfur compound allicin. Restoration of secondary metabolites was more limited, observed only for the saponins alliospiroside A, alliospiroside D, ceposide A, and ceparocide I; the flavonoids quercetin and a quercetin–protocatechuic acid adduct; and four phenolic acids (caffeoylquinic acid, ferulic acid, p-coumaroyl glycolic acid, and vanillic acid). Interestingly, some chemical markers were shared between the control and inoculated Cd-treated samples, including the amino acid leucine, the phenolic acid p-coumaroyl glycolic acid, and the saponins alliospiroside A and alliospiroside D (Fig. 7). However, bacterial inoculation did not restore most of the secondary metabolite markers characteristic of untreated control leaves, such as quercetin 3,4′-O-diglucoside, isorhamnetin 4-O-glucoside, and others (Fig. 7). These findings suggest that *Lb. plantarum* 10CH amendment partially restored the chemical profile of Cd-stressed onion leaves, particularly primary metabolites, shifting it closer to that of the control group.

### Effect of *Allium cepa* L. leaves ethanolic extract on α-glucosidase enzyme

**Fig. 8:**
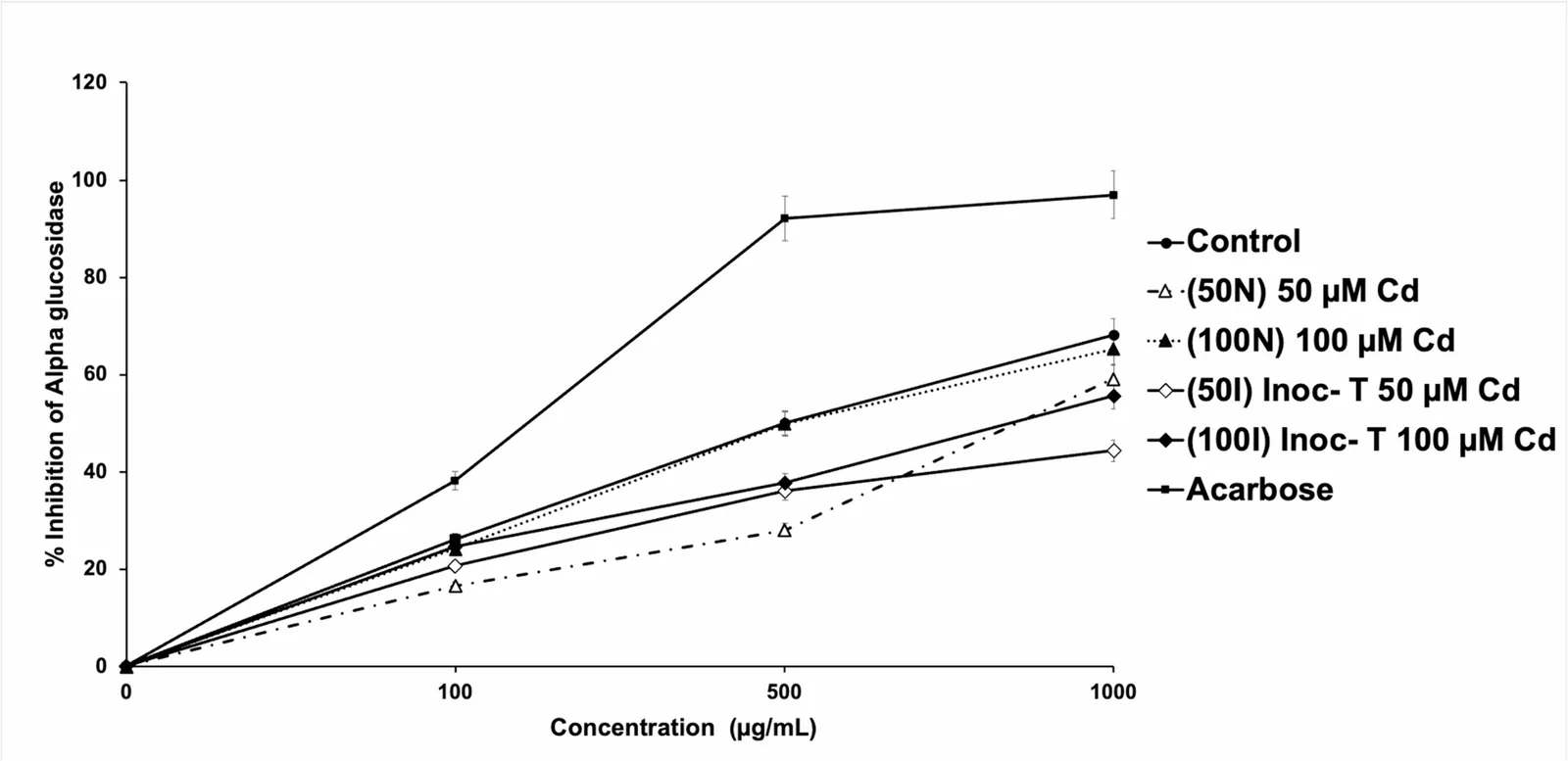
Effect of the investigated *Allium cepa* L. leaves samples on α-glucosidase enzyme. Data are represented as mean ± SD of triplicates.

The investigated onion leaf extracts revealed a dose-dependent α-glucosidase inhibitory effect (Fig. 8). The control samples demonstrated the highest activity, with an IC_50_ of 425.2 ± 0.5 µg/mL, resulting in 68% inhibition at 1 mg/mL concentration. Cd-treated samples showed variable activities. Extracts from plants exposed to 50 µM CdCl_2_ demonstrated moderate inhibition (IC_50_ = 864.3 ± 1.03 µg/mL), whereas those treated with 100 µM CdCl_2_ showed stronger activity (IC_50_ = 466.9 ± 0.09 µg/mL). On the contrary, inoculated samples exhibited weaker α-glucosidase inhibitory potential, with IC_50_ values of 1607 ± 0.21 µg/mL and 848.2 ± 0.78 µg/mL for samples inoculated under 50 µM and 100 µM Cd stress, respectively (Table S2).

### Identification of phytoconstituents responsible for the α-glucosidase inhibitory activity of *Allium cepa* L. leaves samples using an OPLS model

**Fig. 9:**
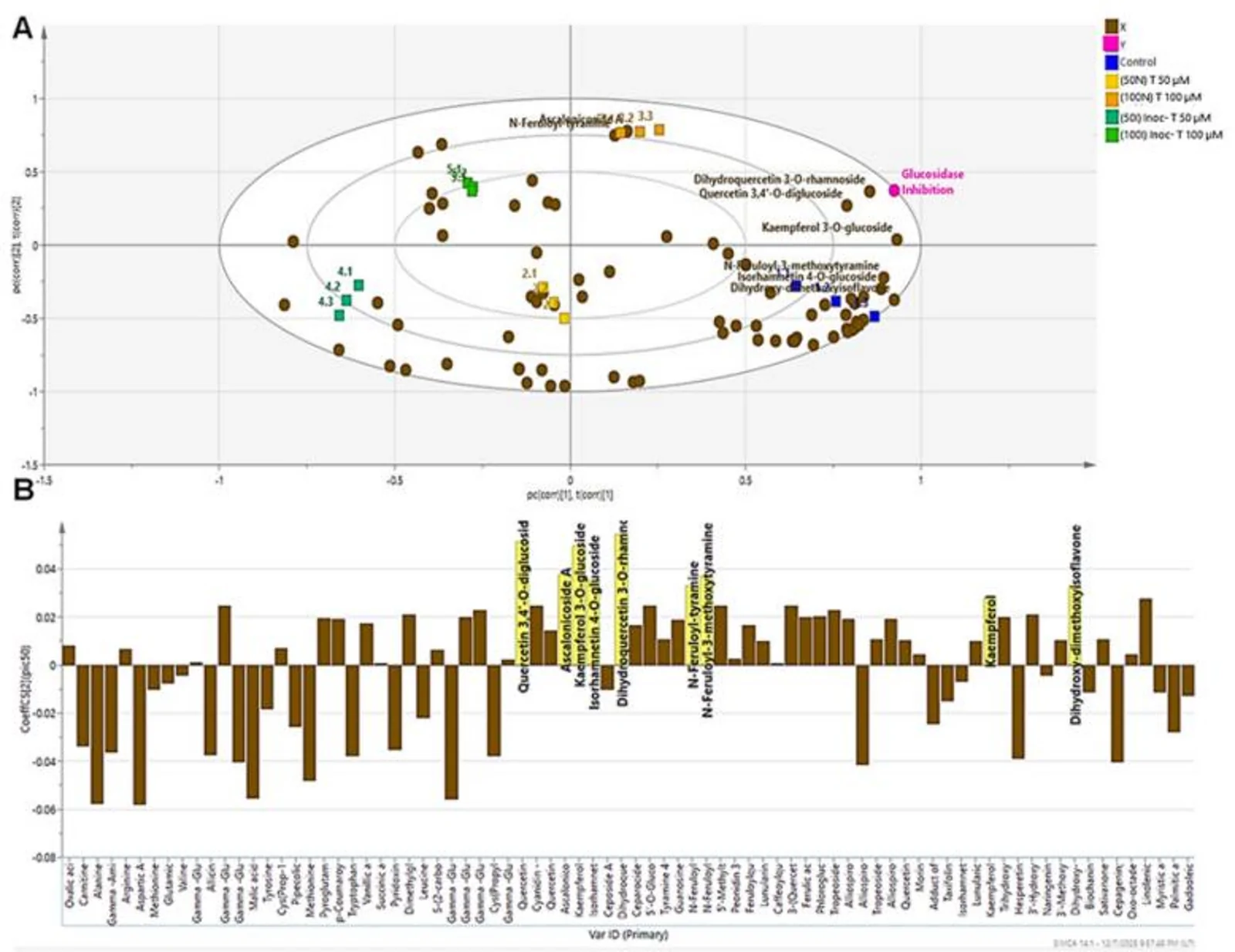
(**A**) OPLS biplot of *Allium cepa* L. leaves samples in correlation to α-glucosidase inhibitory action. (**B**) Correlation coefficient plot of OPLS model showing bioactive metabolities associated with α-glucosidase inhibition.

An OPLS model was constructed (Fig. 9A) to identify putative phytoconstituents correlated to the α-glucosidase inhibitory action of the tested onion samples. The validation parameters (R^2^ = 0.991, Q^2^ = 0.986) demonstrated good model fitness and predictive ability. Model reliability was further supported by permutation testing (20 permutations), which showed no evidence of overfitting (Figure S3). The OPLS model biplot (Fig. 9A) showed the correlation of control untreated onion samples and the samples exposed to 100 µM Cd with the α-glucosidase inhibitory activity. Inspection of the corresponding correlation coefficient plots (Fig. 9B) highlighted several positively contributing metabolites, including the flavonoid glycosides dihydroquercetin 3-O-rhamnoside, quercetin 3,4’-O-diglucoside, kaempferol 3-O-glucoside, and isorhamnetin 4-O-glucoside, as well as the aglycones dihydroxy-dimethoxyisoflavone and kaempferol. Additionally, the saponin ascalonicoside A and the hydroxycinnamic acid amides N-feruloyl-3-methoxytyramine and N-feruloyltyramine were also identified as positively correlated markers contributing to the α-glucosidase inhibitory potential of onion leaves.

## Discussion

Cadmium pollution posses a significant environmental and agricultural threat, directly compromising global food security and crop productivity by substantially reducing both yield and nutritional quality^52–54^. Due to its environmental persistence, Cd is readily taken up by plant root systems and subsequently bioaccumulates in edible tissues. Consequently, crops and vegetables cultivated in Cd-contaminated soils exhibit elevated Cd concentrations, thereby increasing the risk of dietary exposure, heavy metal toxicity, and adverse public health outcomes^55,56^.

The present study demonstrates that cadmium stress caused a significant reduction in *A. cepa* L. growth, as evidenced by decreased root and shoot biomass, which declined further with increasing Cd concentrations. Additionally, there was an increase in the levels of MDA and H_2_O_2_ in *A. cepa*. L. This came in agreement with previous studies describing the growth-inhibitory effects of Cd on plants^57–59^. The negative impact of Cd on plants occurs through disturbing water and nutrient uptake, inhibition of photosynthesis, impairment of mitochondrial function, stimulation of reactive oxygen species production, and membrane damage^60,61^.

Cadmium stress at 50 and 100 µM significantly enhanced the activities of the antioxidant enzymes catalase (CAT), guaiacol peroxidase (GPX), and superoxide dismutase (SOD) (Fig. 4). These enzymes constitute a primary component of the plant defense system against environmental stress. The increase in these enzymes activity indicates their role in scavenging reactive oxygen species (ROS) generated under Cd stress. These findings are consistent with those of Zou et al.^30^, who reported enhanced CAT, GPX, and SOD activities in the leaves and roots of *Allium cepa* var. Agrogatum exposed to 100 µM Cd. In contrast, exposure to 200 µM Cd in our study exerted a negative effect on antioxidant enzyme activities. This decline may be attributed to excessive accumulation of ROS, which subjects the antioxidant system to severe oxidative stress, thereby impairing enzyme functionality^57,62^. In line with this context, previous studies have shown that Cd exposure triggers rapid ROS production, causing cellular damage, inhibiting growth, and damaging genes^63,64^.

The inoculation of *A. cepa* L. with *Lb. plantarum* strain 10CH conferred significant protection against cadmium stress. The observed physiological improvements are consistent with the bacterial predicted profile. Notably, tissue Cd concentrations were significantly reduced by 23% in roots and 22% in leaves, accompanied by a corresponding decrease in the bioconcentration factor (BCF), indicating effective mitigation of Cd uptake and accumulation. Moreover, bacterial inoculation enhanced antioxidant enzyme activities while concurrently reducing reactive oxygen species, including lipid peroxidation products and H_2_O_2_. Collectively, these beneficial effects support the role of *Lb. plantarum* 10CH in alleviating Cd-induced oxidative stress and metal toxicity in plants.

This suggests that *Lb. plantarum* 10CH acts as a barrier to Cd bioavailability or uptake, a role previously attributed to passive biosorption by bacterial cells^65^. A previous study reported the high binding capability and reduction of cadmium by *Lb. plantarum* strain HD 48^66^. Similarly, *Lb. plantarum* CCFM8610 demonstrated potential in alleviating Cd-induced food poisoning, while *Lb. plantarum* MF042018 was proposed as a cost-effective tool for the detoxification of heavy metal-contaminated environments due to its high Cd tolerance and binding efficiency^21^. These findings across diverse *Lb. plantarum* strains suggest the occurrence of conserved genetic determinants that confer cadmium resistance, high binding capacity, intracellular sequestration, and active efflux mechanisms^66^.

Cadmium exposure triggers osmotic stress and subsequent cellular damage in bacteria. To maintain metal homeostasis, bacterial cells tightly regulate metal influx and efflux through specialized ion transport systems that are critical for mitigating cadmium toxicity^67^. Genome sequence analysis of *Lb. plantarum* strain 10CH revealed the presence of genetic determinants supporting its capacity to tolerate and survive under Cd stress. Notably, cadmium-transporting ATPases encoded by the *cadA* gene play a key protective role by functioning as primary efflux pumps that actively export Cd^2+^ across the cell membrane^68^. Moreover, the transcriptional regulator CadC, also identified in the genome, senses intracellular Cd^2+^ levels and derepresses *cadA* expression, thereby establishing a tightly regulated inducible defense system^69^. Furthermore, the predicted *copA* and *copB* genes in the 10CH genome sequence suggest a broader, synergistic efflux capacity for cadmium alongside other heavy metals, including zinc, lead, and copper. The genome also encodes the secondary transporter CzcD, a proton-coupled antiporter that mediates Cd^2+^ efflux in exchange for protons^70^. The ATP-driven CadA pumps and the chemiosmotic-driven CzcD transporter provide a multi-layered defense. This dual strategy is essential for keeping intracellular cadmium levels low and ensuring cell survival under metal stress. In addition, exopolysaccharides (EPS) produced by *Lb. plantarum* play a crucial role in mitigating Cd stress in plants by altering the physicochemical properties of the soil, improving plant growth conditions, and enhancing plant antioxidant capacity. EPS can chelate heavy metals, thereby reducing their bioavailability and delaying uptake by plants, which provides effective protection under heavy metal stress^71^. Furthermore, EPS can induce plant resistance to heavy metals by modulating the lignin biosynthesis pathway, further strengthening the cell wall barrier against Cd^72^.

Investigation of the chemical profiles of *A. cepa* L. revealed that *Lb. plantarum* 10CH bacterial inoculation induced significant shifts in primary metabolism, particularly in amino acid composition and organic acid levels, including succinic acid. This was consistent with previous reports. Infection by the fungus *Stemphylium vesicarium* in *A. cepa* L. significantly increased the levels of various amino acids, including aromatic amino acids like phenylalanine and tyrosine, as well as alanine, leucine, valine, glutamine, and glutamic acid^73^. However, unlike the fungal infection, bacterial inoculation in our study had a beneficial effect, promoting onion plant growth and mitigating cadmium-induced toxicity, as evidenced by the maintenance of normal growth (Fig. 1). Previous reports demonstrated that lactic acid bacterial inoculation of plant species reshapes plant metabolic profiles, with different outcomes according to LAB strain identity, plant species, and organ^74^. For example, Guo et al.^75^ showed that inoculation of alfalfa silage with either *Lactobacillus buchneri* or *Lactobacillus plantarum* had different effects on the plant metabolome: *L. buchneri* inoculation led to increased accumulation of the amino acids gamma-aminobutyric acid, threonine, tyrosine, valine, ornithine, lysine, beta-alanine, and aspartic acid, as well as polyols (2,3-butanediol, glycerol, arabitol, erythritol, mannitol, and threitol), whereas *Lb. plantarum* inoculation resulted in decreased levels of the organic acid succinic acid and the amine cadaverine. In corn, *Lb. plantarum* inoculation altered essential amino acids and volatile organic compounds compared with untreated corn silage, with L-isoleucine and oxoproline being the major free amino acids; bacterial inoculation also markedly increased levels of gamma-aminobutyric acid, pyridoxine, and alpha-linolenic acid^76^. These observations are comparable to our results for the free amino acids aspartic acid, gamma-aminobutyric acid, tyrosine, and valine, which were restored in Cd-stressed onion following bacterial inoculation (Fig. 7C). Regarding secondary metabolites, lactic acid bacterial inoculation of plants has variable effects, with the most commonly reported outcome being an increase in phenolics and flavonoids. *Lb. plantarum* and *Streptococcus bovis* inoculation of triticale has been reported to upregulate plant flavonoid content^77^. Bisakowski et al.^78^ demonstrated that LAB fermentation of red onions increased levels of quercetin monoglycoside relative to quercetin diglycoside existing in untreated controls. Their findings are comparable to our results, where quercetin was among the positively correlated markers in Cd-stressed onion following bacterial inoculation (Fig. 7C).

The examined onion leaf samples exhibited pronounced α-glucosidase inhibitory activity. Such inhibition, along with the suppression of other metabolic enzymes, such as α-amylase and others, represents a well-established therapeutic target in antidiabetic treatment strategies^79,80^. In our study, the control samples showed greater inhibitory activity, exhibiting 68% α-glucosidase inhibition at 1 mg/mL, whereas Schmidt et al.^81^ reported that the bulb of the same red variety achieved 73% inhibition, but only at a much higher concentration of 40 mg/mL. This came in close agreement with Masood et al.^82^, who reported that ethanolic extracts of *A. cepa* L. peel and bulb produced 80% and 40% inhibition, respectively. Their results attributed the α-glucosidase inhibitory effect primarily to the flavonoid and phenolic constituents of the onion peel.

Additionally, our results demonstrated that exposure to 100 µM Cd stress did not markedly alter the α-glucosidase inhibitory activity, as these samples exhibited an IC_50_ of 466.9 ± 0.09 µg/mL, comparable to untreated controls. In contrast, 50 µM Cd stress reduced the inhibitory potency, yielding an IC_50_ value approximately two-fold higher than that of the controls (Table S2). This could be explained by the variability in chemical constituents between the stressed samples under different Cd concentrations (Fig. 5), particularly the differential accumulation of phenolic metabolites. The OPLS model developed in this study indicated that phenolic compounds, especially flavonoid glycosides and their genins, are the main metabolites positively correlated with α‑glucosidase inhibitory activity (Fig. 9). Previous work on Cd toxicity in onion has shown that Cd exposure can modulate phenolic metabolism. Orsuamaez et al. reported that onion bulbs treated with 100 µM Cd had total phenolic content comparable to that of the control, whereas a decline in total phenolics was only observed at Cd levels higher than 100 µM^83^. This is in line with our findings, where treatment with 100 µM Cd increased the accumulation of secondary metabolites, particularly flavonoid glycosides (Fig. 5). This likely explains the strong α‑glucosidase inhibitory activity observed at this concentration. Meanwhile, treatment with 50 µM Cd was associated with reduced α‑glucosidase inhibitory activity, coinciding with lower levels of flavonoid glycosides and aglycones (Fig. 5). These changes suggest that the decline in α‑glucosidase inhibition at 50 µM Cd is linked to decreased levels of key bioactive phenolics.

In addition, bacterial inoculation of onion samples under Cd stress exerted an inverse effect on α-glucosidase inhibition. Inoculated samples displayed two-fold and four-fold increases in IC_50_ values following 100 µM and 50 µM Cd treatments, respectively, relative to the untreated control samples. Several *Lactiplantibacillus plantarum* strains have been reported to exhibit strong in vitro α‑glucosidase inhibitory activity (> 75%), suggesting their strong antidiabetic properties^84^. Furthermore, their hypoglycemic effects have been confirmed in vivo in high‑fat diet and streptozotocin‑induced type 2 diabetic mice, where *Lb. plantarum* administration ameliorated type 2 diabetes^85^. In contrast to these direct probiotic effects, in our study, *Lb. plantarum* 10CH inoculation of Cd-stressed onion leaves altered the plant metabolite profile, leading to increased abundance of amino acids and peptides and relatively lower levels of flavonoid glycosides compared with both untreated control and Cd‑stressed samples (Fig. 5). This shift away from flavonoid glycosides, which were identified by the OPLS model as key metabolites positively associated with α‑glucosidase inhibition, likely underlies the decreased α‑glucosidase inhibitory potential observed in the bacterial‑inoculated, Cd‑stressed samples.

Multivariate data analysis revealed that the α-glucosidase inhibitory activity of onion leaves was positively associated with their flavonoid content, particularly quercetin- and kaempferol-derived aglycones and glycosides (Fig. 9A). Previous studies have demonstrated the antidiabetic properties of quercetin, which significantly inhibited α-glucosidase with an IC_50_ of 0.15 mg/mL^86^. Moreover, its mono- and di-glycosides (quercetin-4′-O-glucoside and quercetin-3,4′-O-diglucoside) have also been reported to exert antidiabetic effects in other *Allium* species^87^. Kaempferol has likewise been identified as an effective α-glucosidase inhibitor, exhibiting an IC_50_ of 11.6 µM^88^. Additionally, in our study, the two hydroxy cinnamic acid amides N-feruloyltyramine and its methoxylated analogue contribute to the α-glucosidase inhibitory activity of *A. cepa* L. leaves. This is consistent with Liu et al.^89^, who reported non-competitive inhibition of α-glucosidase by this class of compounds^90^.

## Conclusion

The present study demonstrates that cadmium stress exerts toxic effects on onion (*A. cepa* L.) plants, manifested by impaired growth, oxidative stress, and modulation of antioxidant enzyme activity, with severe toxicity observed at 200 µM Cd. Inoculation with *Lb. plantarum* 10CH effectively mitigated Cd-induced toxicity by enhancing growth, reducing Cd accumulation and ROS generation, and increasing antioxidant enzyme activities. Furthermore, bacterial inoculation restored primary metabolite levels, including amino acids and organic acids, which were diminished under 100 µM Cd exposure in leaf samples.

Regarding antidiabetic potential, extracts of control onion leaves exhibited strong α-glucosidase inhibitory activity. OPLS analysis identified the flavonoid glycosides as positively correlated with the α-glucosidase inhibitory activity of onion leaf extracts. Cd stress at 50 µM significantly reduced this activity, whereas 100 µM Cd stress increased inhibition relative to the 50 µM treatment. In contrast, *Lb. plantarum* 10CH inoculation negatively impacted the α-glucosidase inhibitory potential of onion extracts. Overall, these results indicate that *Lb. plantarum* 10CH can serve as an effective microbial inoculant to improve onion tolerance to Cd stress up to 100 µM, although it may compromise antidiabetic properties.

Future recommendations include performing inoculation studies using *Lb. plantarum* 10CH alone, without Cd stress, to identify its direct effects on plant growth and metabolism. Detailed soil characterization is also recommended to better understand how soil properties and the presence of other beneficial bacteria influence the Cd-bioremediation action of the strain. Furthermore, since the present study focused on leaf-level metabolic responses, future work should extend chemical profiling to other onion organs, including roots and bulbs, under Cd stress and following *Lb. plantarum* 10CH inoculation, to provide a more comprehensive understanding of the whole plant metabolic response. Further studies on other *Lactiplantibacillus* species are required to identify strains with broader Cd tolerance that can be applied for bioremediation of heavy-metal-contaminated onion crops.

## Supplementary Information

Below is the link to the electronic supplementary material.

Supplementary Material 1

## Data Availability

All data supporting the findings of this study are available within the paper and its Supplementary Information.
