# Supplementary material for: Chemometric insights into Lactiplantibacillus plantarum effects on onion (Allium cepa L.) metabolism and antidiabetic activity under cadmium stress
Source: Sci Rep. 2026 Sep 5;16:27823. doi: 10.1038/s41598-026-67251-0 (PMC13546298; doi:10.1038/s41598-026-67251-0)
Supplement: Supplementary file 1 [file 41598_2026_67251_MOESM1_ESM.docx]

**Supplementary Information**

**Chemometric Insights into *Lactiplantibacillus plantarum* Effects on Onion (*Allium cepa* L.) Metabolism and Antidiabetic Activity under Cadmium Stress**

**Salwa M. Abdel Rahman^1^, Nancy M. El Halfawy^1^, Rahma S. R. Mahrous^2, *^**

**^1^**Department of Botany and Microbiology, Faculty of Science, Alexandria University, Alexandria, Egypt

**^2^**Department of Pharmacognosy, Faculty of Pharmacy, Alexandria University, Alexandria, Egypt

***Corresponding author:**

Rahma S. R. Mahrous*

[Rahma.mohammed@alexu.edu.eg](mailto:Rahma.mohammed@alexu.edu.eg)

Address: 1 El-Khartoum Square – Azarita, Tel: +2 01001223411, Fax: 4873273-4871668, Postal Code: 21521

**Salwa M. Abdel Rahman:** https://orcid.org/0000-0003-3640-2039

**Nancy M. El Halfawy:** https://orcid.org/0000-0002-5687-0158

**Rahma S. R. Mahrous:** <https://orcid.org/0000-0003-4220-9796>

 **Figure S1. UPLC-MS chromatograms of *Allium cepa* L. leaf samples in positive ion mode: untreated control (0 μM Cd), Cd-stressed at 50 μM and 100 μM, and Cd-stressed samples inoculated with *Lb. plantarum* 10CH at 50 μM and 100 μM.**

**Figure S2. Principal component analysis (PCA) score scatter plot of *Allium cepa* L. leaves samples.**

**Table S1. BLASTp analysis of heavy metal tolerance genes predicted in *Lb. plantarum* 10CH genome sequence.**

| **Gene** | **Product** | **Length (aa)** | **Top Hit Accession** | **Query Coverage (%)** | **Identity (%)** | **Amino acid sequence** |
| --- | --- | --- | --- | --- | --- | --- |
| *cadA* | Probable cadmium-transporting ATPase (EC 3.6.3.3) | 634 | WP_047674272.1 | 100 | 100 | MKFQLFLTKHTNQITLVTGILIVLGMLSKYLLQFTLGYQVILAVASIIAVIPIAVRAWSA  LRNKVFSIELLVSIAVIGAFIIGEFNESAIVTFLFLFGSYLESKTLQKTRTAIKGLTDMS  PTTATLVTDDGTEEVDVDDVDEGDVVLVKTGSQVPADGIVVEGNGYLNEASITGEARQIS  KQLDDSVYSGTMVENGYLKIKATQVGDDTTFAKIIELVEEAQDTKSKAEKFIDRFAQYYT  PAVLVLAVLVFAFSRDFRLAITVLVLGCPGALVIGAPVSNVAGIGNGAKRGILIKGGEVV  DTFAKVDTLVFDKTGTLTEGNTAVTTMHTYTNNADNQLALAAAIEGVSDHPLGQAIVSYA  DQQSAGVAPLLDDTETVKGQGICAQVGKQEVVIGNQKMLTAHNIKLNPTQLKDLNDLQAG  GQSTVIMAVDGQVQLIFGIADTIRPGVKDSLAALKAQGIKKLVMLTGDNELTAQAVANEL  NLDEVHANLLPEEKVEYVKKLKAAGNTVAFIGDGINDSPSIANADIGIAMGSGTDVAIDT  SDVVLMQSSFPALVHAHGLAKKTVLNTRENIFIAIATVAFLLIGLIFGYIYMASGMLVHE  ASILVVIFNAMRLINFQTKFDKHQPTKTIQAATA |
| *czcD* | Cobalt/zinc/cadmium efflux system component | 320 | WP_047673362.1 | 100 | 100 | MSIQRLPDWHQIEQPVVISLKTARHHLWGNVAVYLGLFIIELLGARWGHSQALRADAFNN  LSGIFSTGLLMTGLFIASRTHDDDLFGAPIAPAEQQQLGPRIQQSRFRFETIFNLLAGTI  MAAIGIDIIIKGVMTFATRTPFKNLSSIAGLAAGFSFVVLLVLWAFNHYWSRKLNNAALT  AASRDTFTDALTSLTTVLTILVTVKLHLQWFDNLISIGLGLYILNTGTKIFRESSLNLVD  YFDPYLEDRYQRRIADLTSVRKVIFLKAHYDGNLIMLRATIVVPPRMTAIEITRLIETIN  QIMWQEFGVMETDVMITTSY |
| *czcD*2 | Cobalt/zinc/cadmium efflux system component  CzcD | 295 | WP_099745986.1 | 100 | 99.66 | MAHSHQIQQQTSAFKIGVALNTAFIVLEAGYGFASGSLALVADAGHNLSDVLGLLISWLA  IWLGQKSANDKYTYGYKSSSILAALFNAVFLLVAIGAISVEAIQRLSNPGPVAEWDVIIV  AAVGVVINGFTALLFMKGQKHDLNIKGAFLHMAADAGVSIGVVIAGFIILKTGWFWLDPV  VSLLIAVVILIGTWGLLRDAMNYSLEAVPNNVDRQAIADYLTSQPTVNQIHDLHIWGMST  TETAMTVHLCRSTLADNNAFLEQLNKDLSAQFPLTHITIQIELGKVDYETTDSSI |
| *copA* | Copper-translocating P-type ATPase (EC 3.6.3.4) | 641 | WP_046040517.1 | 100 | 100 | MSTTKRFWISLIFSLPLLANMILMPFGWMLPGGDWTQLILTTIIMAVSARPFLQSAWASF  TKHHANMDTLVAIGTATAYFYSLYAMATGQAVFFESAALVTTFVLLGQVFEERMRHNASS  AVAKLVGLQAKEAEVLRDQEFVKVPLDQVVVGDIIRVKPGEKIPVDGVITTGRSTVDESM  VTGESMPVQKTVADAVIGSTINSNGTFQFKASKVGDQTMLAQIVELVKKAQNSHAPIQKL  TDKVSDIFVPIVLIIAIVTFLTWYVFLNASLANALIFAVAVVVIACPCALGLATPTALMV  GTGRGAKMGILIKNGEVLEAVNDVKTVIFDKTGTITVGHPQVTDVIGDEAQVLRVATSLE  AASEHPLAAAILKRGTARGIQAPTATNFQAIEGKGVTAQVEGQLAVVGNHKLLTDEPISA  ELQRQATELQDAAKTVVYVGLNHEIIGLIAIQDVAKSTSEVAIASLKARGLKTVMLTGDN  ERVAQAVADQVGIDEVIADVLPGDKAEHVHAFQKAGKVAFVGDGINDAPALTTADVGIAM  GSGTDIAIDAGGIVLVKNDLRDVDRALALSKKTFNRIKLNLFWAFVYNVLGIPVAAGLFF  AIGLTLSPELAGLAMAFSSLSVVTSSVLLNKAKISSNAQPA |
| *copB* | Copper-translocating P-type ATPase (EC 3.6.3.4) | 679 | WP_047674245.1 | 100 | 100 | MKDMDMQHMAHHDSNDMGDMDMGNGEMMHMGNLKLKFWVALVLMIPIIIMSPMMGVNLPF  QIVFRPWSDYIVAILGTILFLYGGQPFFSGAKAEVKNHKPAMMTLITMGISVAYVYSIYA  VVANDLLNAKPMVTDFFWELATLIVIMLLGHWIEMNTVMNAGSAVDKLAKLLPSTAHKLV  DGQVHDVAIADLANGDQVQVRAGEQIPADGTVLNGATSVNEAMVTGEAKLVEKRAKDQVV  GGSVNGDGTFEMKVTGTGESGYLAQVMKLVSDAQAAKSSQENMADKVAGLLFYAALTVAI  VAFIVWLLVGNLALALSIAVTVLVIACPHALGLAIPLVVARSTAIAASHGLLIRNRDAME  QVKKLNYALMDKTGTLTAGNFKVADYQSSADRLTADAVLAMMAGLENGSSHPLAVGILAA  AKEHQLTPKVSQDVHQITGVGLSGTIDGQTYQIVSAAYLDKQNLAYDQQTFNRLASAGNS  VSFLIQEQTVLGFVAQGDQIKPEAKNLIDSLKAQNITPVMLTGDNPQSAQVVAKQLGITE  VHAGLLPEDKAKLVQQYQADGSHVMMIGDGVNDAPSLARADIGVAIGSGTDVAIDSADIV  LVKSNPNDVVDFLALARRTTRKMAQNLWWGAGYNVITIPLAAGILAPLGFILNPMFGAVV  MSLSTVIVAINAMTLRLKD |
| *cadA* | Lead, cadmium, zinc and mercury transporting ATPase (EC 3.6.3.3) (EC 3.6.3.5) | 614 | WP_047674235.1 | 100 | 100 | MQHYYKLILTVSVGIIAVILQFGLHYQLAAQIIITLMGSLMALSMLVEMIKTLRSGKYGV  DLLAITAIVATLAVGEYWASLVVLIMLTGGDSLEDYAAKRANTELKALLDNSPRIAHRSV  AGQLTDIAVEDVQIGDQLVVKPGELVPVDGHLIQGTALFDESSLTGESKPVDKHVGDDIM  SGAVNGDSAVTMVVDKLAIDSQYQQLVKLVKESESRPAKFVRLADRYAVPFTLVAYVIAG  VAWAFSGDPHRFAEVLVVASPCPLILAAPVALVSGMSRTSRNGIVVKTGDMLEKLSTAKS  AAFDKTGTITSGQLTVNQIVPQPGFTTEQILHLAASAEQNSSHILARSLVKYASATSLSP  ATQLAEVTGNGVTATIEQQQVKVGKLKFVAPTTTAKPLATTAIYVGIDDQYAGYITFIDN  VRPEAAATLQALHAEGVQNVMMLTGDQQAIADHIAADVGIDTVAADLLPADKIAHLKAVP  AAERPVIMVGDGVNDAPSLAVADVGIAMGAHGSTAASESADVVILKDDLSRVVTAVRIAK  DTMQVAKQAVWIGIAICTILMLIASTGIIPALFGAMLQEVVDTVSILWALRALRDRPQSS  VSSTTTTPITKDVS |
| *cadC* | Cadmium efflux system accessory protein | 123 | APR08799.1 | 100 | 99.19 | MLSNKDTCEIYCYDEKKVNRIREELQKEDISSVSQLFKALADENRAKITYALCQDGELCV  CDVANIIGSTVATASHHLRTLHKQGIVKYRKEGKLAFYSLDNEYIKQLILIALAHKNEVK  ANV |
| *cadA/zntA* | Cadmium-transporting ATPase (EC 3.6.3.3) | 709 | WP_076638566.1 | 100 | 99.86 | MSDQQAKLSEEEMKTYRVQGFTCTNCAGIFENNVKQLPGVKDAKVNFGASKVYVQGNTTI  EELEKAGAFENLKVRDEKEQKVEREPFWKQKENIKVYISALLLVISWFLGEQLGEEHLLP  MIGYAASILIGGYTLFIKGLKNLSRLKFDMNTLMTIAIIGAAIIGKWGEGATVVILFAIS  EALERYSTDKARQSIGSLMDIAPKEALIRRGNEEMMIHVEDIQVGDIMIVKPGQKLAMDG  LVIKGTSTLNQAAITGESVPVTKTIDDEVFAGTLNEEGLLEVKVTKRDEDTTISKIIHLV  EEAQAERSPSQAFVDKFAKYYTPAIVLLALLIAVVPPLFGGDWSQWLYQGLAVLVVGCPC  ALVVSTPVAVVTAIGNAAKNGVLIKGGIHLEEAGHLKAIAFDKTGTLTKGIPAITDIVTY  GGNENELLTVTAAIEKGSQHPLASAIVRKAEENGLNLNGVSVEEFQSITGKGVKAKVNNE  MYYVGSPNLFEELHQTIESTIKERITRMQTEGKTVMVLGTEKEILSLIAVADEMRETSKE  VISKLNHIGIETVMLTGDNQRTAEAIGKQVGVSDIKADLLPEDKLNFIKELLGKHQSVGM  VGDGVNDAPALAASSVGVAMGGAGTDTALETADIALMADDLSKLPYTIKLSRKALAIIKQ  NITFSLAVKLVALLLVMPGWLTLWIAIFADMGATLLVTLNSLRLLKVKG |

**Table S2. Effect of the investigated *Allium cepa* L. leaves samples (control, Cd-treated, and inoculated with *Lb. plantarum* 10CH following Cd stress) on α-glucosidase enzyme.**

| **Sample** | **IC_50_ (µg/mL)^#^** |
| --- | --- |
| Control | 425.2 ^b^ ± 0.5 |
| 50 µM Cd-treated | 864.3*^, d^ ± 1.03 |
| 100 µM Cd-treated | 466.9*^, c^ ± 0.09 |
| Inoculated 50 µM Cd-treated | 1607*^, e^ ± 0.21 |
| Inoculated 100 µM Cd-treated | 848.2*^, d^ ± 0.78 |
| Acarbose (positive control) | 130.6*^, a^ ± 0.06 |

\# Data are mean ± SD of triplicates. **p* < 0.05 indicates a statistically significant difference compared to control untreated extract. Different letters indicate statistically significant at *p* < 0.05 as evaluated by Tukey’s HSD post-hoc.

**Figure S3. Permutation plot of OPLS model using 20 permutations.**
